# Inactivation of the Euchromatic Histone-Lysine *N*-Methyltransferase 2 Pathway in Pancreatic Epithelial Cells Antagonizes Cancer Initiation and Pancreatitis-Associated Promotion by Altering Growth and Immune Gene Expression Networks

**DOI:** 10.3389/fcell.2021.681153

**Published:** 2021-06-23

**Authors:** Guillermo Urrutia, Thiago Milech de Assuncao, Angela J. Mathison, Ann Salmonson, Romica Kerketta, Atefeh Zeighami, Timothy J. Stodola, Volkan Adsay, Burcin Pehlivanoglu, Michael B. Dwinell, Michael T. Zimmermann, Juan L. Iovanna, Raul Urrutia, Gwen Lomberk

**Affiliations:** ^1^Division of Research, Department of Surgery, Medical College of Wisconsin, Milwaukee, WI, United States; ^2^Genomic Sciences and Precision Medicine Center, Medical College of Wisconsin, Milwaukee, WI, United States; ^3^Department of Pathology, Koç University Hospital, Istanbul, Turkey; ^4^Department of Pathology, Adiyaman University Training and Research Hospital, Adiyaman, Turkey; ^5^Department of Microbiology and Immunology, Medical College of Wisconsin, Milwaukee, WI, United States; ^6^Center for Immunology, Medical College of Wisconsin, Milwaukee, WI, United States; ^7^LaBahn Pancreatic Cancer Program, Medical College of Wisconsin, Milwaukee, WI, United States; ^8^Department of Biochemistry, Medical College of Wisconsin, Milwaukee, WI, United States; ^9^Clinical and Translational Sciences Institute, Medical College of Wisconsin, Milwaukee, WI, United States; ^10^Centre de Recherche en Cancérologie de Marseille (CRCM), INSERM U1068, CNRS UMR 7258, Aix-Marseille Université and Institut Paoli-Calmettes, Parc Scientifique et Technologique de Luminy, Marseille, France; ^11^Department of Physiology, Medical College of Wisconsin, Milwaukee, WI, United States; ^12^Department of Pharmacology and Toxicology, Medical College of Wisconsin, Milwaukee, WI, United States

**Keywords:** pancreatic carcinoma, epigenomics, histone methyltransferases, gene regulatory networks, tumor microenvironment

## Abstract

Pancreatic ductal adenocarcinoma (PDAC) is an aggressive, painful disease with a 5-year survival rate of only 9%. Recent evidence indicates that distinct epigenomic landscapes underlie PDAC progression, identifying the H3K9me pathway as important to its pathobiology. Here, we delineate the role of Euchromatic Histone-lysine *N*-Methyltransferase 2 (EHMT2), the enzyme that generates H3K9me, as a downstream effector of oncogenic KRAS during PDAC initiation and pancreatitis-associated promotion. *EHMT2* inactivation in pancreatic cells reduces H3K9me2 and antagonizes Kras^*G*12*D*^-mediated acinar-to-ductal metaplasia (ADM) and Pancreatic Intraepithelial Neoplasia (PanIN) formation in both the *Pdx1-Cre* and *P48*^*Cre/+*^ *Kras*^*G*12*D*^ mouse models. *Ex vivo* acinar explants also show impaired EGFR-KRAS-MAPK pathway-mediated ADM upon *EHMT2* deletion. Notably, Kras^*G*12*D*^ increases EHMT2 protein levels and EHMT2-EHMT1-WIZ complex formation. Transcriptome analysis reveals that *EHMT2* inactivation upregulates a cell cycle inhibitory gene expression network that converges on the *Cdkn1a/p21-Chek2* pathway. Congruently, pancreas tissue from *Kras*^*G*12*D*^ animals with *EHMT2* inactivation have increased P21 protein levels and enhanced senescence. Furthermore, loss of *EHMT2* reduces inflammatory cell infiltration typically induced during Kras^*G*12*D*^-mediated initiation. The inhibitory effect on Kras^*G*12*D*^-induced growth is maintained in the pancreatitis-accelerated model, while simultaneously modifying immunoregulatory gene networks that also contribute to carcinogenesis. This study outlines the existence of a novel KRAS-EHMT2 pathway that is critical for mediating the growth-promoting and immunoregulatory effects of this oncogene *in vivo*, extending human observations to support a pathophysiological role for the H3K9me pathway in PDAC.

## Introduction

Pancreatic cancer remains a devastating disease, currently ranking 3rd for cancer-related deaths in the United States and predicted to rank 2nd by 2030 (Rahib et al., 2014). Pancreatic ductal adenocarcinoma (PDAC), the most common type, arises through a stepwise progression from low-grade to high-grade Pancreatic Intraepithelial Neoplasia (PanINs) and eventually leading to invasive adenocarcinoma (Andea et al., 2003). The transitions through increasingly aggressive lesions are accompanied by accumulation of genetic and epigenetic alterations (Macgregor-Das and Iacobuzio-Donahue, 2013; Lomberk et al., 2019). Activating mutations in the *KRAS* gene are almost consistently the initiator and present even in low-grade PanINs (Hezel et al., 2006). As a result, oncogenic KRAS, in particular Kras^*G*12*D*^, serves as the cornerstone of genetically engineered mouse models (GEMM) for PanIN lesions, as well as PDAC, when crossed to additional genetic models (Herreros-Villanueva et al., 2012). Activation of a *Kras*^*G*12*D*^ allele in mice induces hyperplasia, acinar-to-ductal metaplasia (ADM), and PanIN formation (Hingorani et al., 2003). Given the lack of additional genetic events prior to higher-grade PanIN lesions, these transitions from hyperplasia and ADM through establishing low-grade PanIN are thought to occur at an epigenomic level (Shibata et al., 2018). In addition, evidence supports that the heterogeneity of PDAC results from the presence of distinct epigenomic landscapes, which also carries the potential to induce certain types of plasticity between PDAC subtypes (Lomberk et al., 2018). Therefore, dissecting the role of various epigenomic pathways should provide important insights into PDAC tumorigenesis.

Histone H3 lysine 9 (H3K9) methylation is emerging as an important transcriptional regulatory and epigenetic pathway in pancreatic cancer (McDonald et al., 2017; Lomberk et al., 2019). Histone lysine methylation plays a critical role, along with DNA methylation, for long-term epigenetic maintenance, as well as propagation of overall chromosome structural features and stability (Sims et al., 2003; Martin and Zhang, 2005). Euchromatic histone-lysine *N*-methyltransferase 2 (EHMT2/G9a) is the main SET domain-containing histone lysine methyltransferase (HMT) responsible for catalyzing H3K9 mono- and di-methylation (H3K9me1 and H3K9me2) (Casciello et al., 2015). The fact that EHMT2 functions in the regulation of various processes, including cellular differentiation, proliferation, epithelial-to-mesenchymal transition (EMT), senescence, DNA replication and DNA repair among others, has suggested a key role for this protein in the epigenetics of human cancers (Shinkai and Tachibana, 2011; Dong et al., 2012; Casciello et al., 2015; Yang et al., 2017). Indeed, EHMT2 is upregulated in many cancers, including PDAC (Casciello et al., 2015), in which its expression correlates with shorter times to relapse and survival (Pan et al., 2016). Pharmacological inhibition of EHMT2 in the PANC-1 PDAC cell line has implicated potential roles in senescence, autophagy and overcoming chemotherapy resistance (Yuan et al., 2012, 2013; Pan et al., 2016). However, the impact that its function has on the effect of activated oncogenes, such as KRAS, during PDAC development *in vivo* remained to be defined and constitutes the main goal of the current study. Initial studies in *P48*^*Cre/+*^ *Kras*^*G*12*D*^ mice showed that *EHMT2* deficiency modifies PanIN progression and animal survival (Kato et al., 2020). The current study uses several *in vivo* along with *in vitro* models to extend mechanistic information on the impact of EHMT2 in *Kras*^*G*12*D*^-mediated initiation. In addition, we also study the role of this protein in the context of inflammation-associated pancreatic cancer using pancreatitis-induced neoplastic promotion. We provide new mechanistic information that supports the dysregulation of gene expression networks antagonistic to cell growth and induction of P21/β-galactosidase-positive senescence. We also report that oncogenic KRAS regulates EHMT2 levels and the formation of its complex with EHMT1 and WIZ, leading to increased levels of its enzymatic product, the H3K9me2 mark. We find reduced inflammatory responses in pancreas tissue from animals carrying *EHMT2* inactivation compared to *Kras*^*G*12*D*^ mice retaining the wildtype allele, which is also reflected by functionally coherent gene expression networks. Moreover, genetic inactivation of *EHMT2* sustains the antagonistic effects on initiation of *Kras*^*G*12*D*^-driven neoplastic cell growth even in the pancreatitis-associated promotion model. This phenotypic response is accompanied by alterations in both cell growth and immunoregulatory gene expression networks, thereby affecting the tumor microenvironment through disruption of this pathway in epithelial cells. Combined, these data designate EHMT2 as part of the *Kras*^*G*12*D*^ pathway, as well as reveal a role for this epigenetic regulator in the growth-promoting effects and inflammatory response necessary for Kras^*G*12*D*^-induced carcinogenesis.

## Materials and Methods

### Cell Lines and Reagents

Mouse iKras 4292 cell lines were obtained from Dr. Marina Pasca di Magliano [University of Michigan](Collins et al., 2012). Cells were maintained in RPMI 1640 media supplemented with 10% (v/v) FBS and 10 μg/ml doxycycline hyclate (doxy, Sigma Aldrich, Cat# D9891) for regular culture. For Kras^*G*12*D*^ induction experiments, cells were deprived of doxy at least 48 h for beginning experiments (0 h, baseline). Then, fresh media containing doxy 10 μg/ml was added, and cells maintained under culture during multiple time intervals (12, 24 and 48 h). Human hTERT-E6/E7-HPNE (ATCC Cat# CRL-4037, RRID: CVCL_C468) and hTERT-E6/E7-HPNE-KRAS^*G*12*D*^ (ATCC Cat# CRL-4039, RRID: CVCL_C470) pancreatic cell lines were obtained from ATCC and maintained in appropriate media according to recommendations. All cells were cultured at 37°C in a humidified incubator with 5% CO2 and were used to a maximum of 30 passages. All cell lines tested negative for Mycoplasma with last testing performed in July 2020.

### Western Blot Analysis

Mouse pancreas (∼100 mg) was cut in small pieces and homogenized in protein extraction buffer [10 mM Tris–HCl, 1 mM EDTA, 1% (v/v) Triton-X100, 1% (w/v) Sodium Deoxycholate, 0.1% (w/v) SDS, 140 mM NaCl and 1 mM PMSF] with freshly added protease and phosphatase inhibitors (Thermo Fisher Scientific). Homogenized tissue was moved to a 2 mL microcentrifuge tube containing 1 stainless steel bead (7 mm diameter) and further dissociated using TissueLyser LT (2 min at 50 Hz). The lysate was then sonicated twice (amplitude 5 for 10 s) and supernatant was cleared by centrifugation (10,000 rpm, 10 min, 4°C). HPNE and iKras proteins were collected from cells by lysis in protein extraction buffer supplemented with phosphatase and protease inhibitors. Equal amounts of protein from pancreas or cell lysates were electrophoresed on 12% SDS-PAGE gels and transferred to nitrocellulose membranes (GE Healthcare). Membranes were blocked in either 5% (w/v) milk or 3% (w/v) BSA for 1 h, and primary antibody incubations were performed overnight at 4°C with rocking. Supplementary Table 1 contains primary antibodies used. Anti-mouse or anti-rabbit HRP-conjugated secondary antibodies (1:5000, Millipore) were incubated on the membranes for 1 h at room temperature with agitation, followed by detection with chemiluminescence (Thermo Fisher Scientific). Quantification of bands in *n* = 3 experiments was completed using ImageJ and statistical significance determined by Student’s *t*-tests in GraphPad Prism 7.

### Immunofluorescence, Proximity Ligation Assays (PLA) and Confocal Microscopy

iKras 4292 cells were stimulated with doxy at the indicated intervals, fixed using 4% Formaldehyde and washed three times with 1× PBS. Cells were permeabilized using 0.2% Triton X-100 for 10 min, PBS washed and blocked with 1% (w/v) BSA in PBS-Tween 20 solution. Slides were then incubated with EHMT2, EHMT1 and WIZ primary antibodies at 4°C overnight and labeled using Alexa Fluor-conjugated secondary antibodies. For proximity ligation assays (PLA) assays, the Duolink PLA Starter Kit (Sigma Aldrich, Cat# DUO92102) was used according to manufacturer’s instructions. Coverslips were mounted using Prolong Gold antifade mounting media (with DAPI, Life technologies, Cat# P36930). Antibodies used are listed in Supplementary Table 1. Images were acquired using a Zeiss LSM510 confocal microscope with a 63× magnification objective.

### Immunoprecipitation (IP) and Mass Spectrometry

Immunoprecipitation (IP) of EHMT2 was performed by conjugating the EHMT2 antibody (Thermo Fisher Scientific Cat# PA5-34971, RRID: AB_2552320) or control IgG antibody to Protein A/G Magnetic Beads (Thermo Fisher Scientific) through disuccinimidyl suberate crosslinking. iKras 4292 cells were grown and then stimulated with doxy for 24 h. Cells were lysed with IP Lysis/Wash Buffer (Thermo Fisher Scientific-Pierce), and lysates were incubated overnight with the antibody conjugates at 4°C. Immunoprecipitated complexes were washed, eluted, and run on a 4–15% Criterion^TM^ Tris–HCl Protein Gel (Bio-Rad). The gel was subsequently visualized with BioSafe^TM^ Coomassie Stain (Bio-Rad). Each gel lane was de-stained, dehydrated, dried, and subjected to trypsin digestion. Subsequently, liquid chromatography (LC)-ESI-MS/MS analysis was performed on a Thermo Scientific LTQ Orbitrap mass spectrometer at the Mayo Clinic Proteomics Core.

### Mouse Breeding, Genotyping and Caerulein Treatment

Animals were housed in standard mouse housing with controlled temperature, humidity and light cycles and provided with *ad libitum* standard rodent chow and water. Mice were euthanized using CO_2_ following institutional guidelines. Tissues were collected and preserved in formaldehyde for 24 h and then moved to 70% (v/v) ethanol for histological analysis or freshly processed for cellular studies. *B6.129S4-Kras*^*TM* 4*Tyj*^/*J* (*LSL-Kras*^*G*12*D*^, IMSR Cat# JAX:008179, RRID: IMSR_JAX:008179) (Jackson et al., 2001), *B6.FVB-Tg(Pdx1-Cre)6Tuv/J* (*Pdx1-Cre*, IMSR Cat# JAX:014647, RRID: IMSR_JAX:014647) (Hingorani et al., 2003), *Ptf1a*^*TM* 1(*cre*)*Hnak*^/*RschJ* (*P48*^*Cre/+*^, IMSR Cat# JAX:023329, RRID: IMSR_JAX:023329) (Nakhai et al., 2007), and *B6.Cg-Tg(CAG-cre/Esr1^∗^)5Amc/J* (*CAGGCre-ER*^TM^, IMSR Cat# JAX:004682, RRID: IMSR_JAX:004682) (Hayashi and McMahon, 2002) were purchased from Jackson Laboratories. *EHMT2 flox/flox* (*EHMT2*^*fl/fl*^) animals were a generous gift received from Dr. Oltz (Tachibana et al., 2007). All animals have been described previously and were maintained on a C57Bl/6 background. *EHMT2*^*fl/fl*^ mice were crossed with animals containing *LSL-Kras*^*G*12*D*^, *Pdx1-Cre*, *P48*^*Cre/+*^ or tamoxifen-inducible (*CAGGCre-ER*^TM^) Cre drivers. Animals were weaned after 3 weeks and 0.4–0.8 cm length of tail was taken for genotyping. DNA was extracted from the tail pieces using Qiagen’s DNeasy^®^ Blood and Tissue kit (Cat# 69506). PCR was performed as previously described (Tachibana et al., 2007) with products run out an 2.5% agarose gel. Animals that were used in aging studies with no additional treatments were sacrificed at 4, 6, or 8 weeks.

To induce chronic pancreatitis, caerulein was administered via IP injection to 4-week-old mice once a day, five times a week, for a total of 4 weeks at a dose of 50 μg/kg. Animals were given 7 days with no treatment before sacrifice to allow recovery. Animal and pancreas weights were recorded at the time of sacrifice and tissue was taken for RNA, protein and histological analysis.

### Immunohistochemistry

Immunohistochemistry was performed on mouse pancreas tissue as described previously (Mathison et al., 2013). Primary antibodies (Supplementary Table 1) were incubated overnight at 4°C. Slides were developed with Nova Red (Vector Laboratories, Cat# SK-4800) and counterstained with Mayer hematoxylin. Five random fields (10× objective) per slide, containing at least 1,000 cells per field, were imaged, and counted.

### 3D Acinar Culture

*CAGGCre-ER*^TM^ and *CAGGCre-ER*^TM^;*EHMT2*^*fl/fl*^ mice were euthanized and pancreas immediately injected with 2 mL of chilled collagenase P (1.33 mg/ml), dissected and cut into small pieces for digestion (20 min at 37°C with gentle shaking). Acinar cells were collected by centrifugation and resuspended in 3D culture base medium (RPMI 1640 medium supplemented with 1% FBS, 0.1 mg/ml soybean trypsin inhibitor, sodium pyruvate 1× and antibiotics). 96-well plates were pre-coated with Matrigel (Corning, Cat# 356231, 30 μL) for 1 h at 37°C. Cell suspensions were mixed 1:1 with Matrigel and plated (100 μL/well). The cell/Matrigel mix was allowed to solidify for 1 h at 37°C before addition of 175 μL 3D culture media. For the first 48 h, DMSO or 4-OHT (10 mmol/L) was added, followed by EGF (20 ng/mL) or TGFα (50 ng/mL) for an additional 5–6 days (Martinelli et al., 2016). Pictures from ductal structures were taken after 8 days in culture using a Canon EOS Rebel Xsi camera mounted on a Nikon Eclipse TS100 microscope at 10× magnification. Duct size was measured using ImageJ software, and plotted values represent fold-change of treated groups over respective controls.

### RNA Extraction

For RNA extraction from tissue, a section of pancreas was taken at time of euthanasia, minced in RNAlater and snap frozen with liquid nitrogen. RNA extraction was completed with ToTALLY RNA Kit (Ambion, Cat# AM1910) according to manufacturer’s protocols. Briefly, frozen samples were lysed with tissue Denaturation Solution and disrupted in the TissueLyser LT (Qiagen, 2 min at 50 Hz) with a single, 7 mm stainless steel bead. RNA isolation continued with Phenol:Chloroform:IAA and Acid-Phenol:Chloroform extractions to the aqueous phase and a final isopropanol precipitation. Precipitated RNA was cleaned up according to Qiagen’s protocol on the miRNeasy column (Qiagen, Cat# 217004), with an on-column DNA digestion and RNaseOUT (Invitrogen, Cat# 10777019) added to final elution to reduce degradation during storage at −80°C. Isolation of RNA from cells was completed according to Qiagen’s protocol for the RNeasy kit, including an on-column DNase digestion.

### RNA-seq and Bioinformatic Analysis

RNA was quantified by Qubit (Invitrogen) and quality assessed with a Fragment Analyzer (Agilent), with the highest quality (typically RINs > 6, DV200 > 80%) utilized for library preparations. Pancreas RNA was prepared and sequenced in the Mayo Clinic Medical Genomics Core using the Illumina TruSeq RNA v2 library preparation kit and the Illumina High Seq-2000 with 101 bp paired end reads. Reads were aligned to the mouse reference transcriptome Gencode vM23 (GRCm38.p6) with at least 24 million mapped read pairs acquired per sample. Sequencing reads were processed through the GSPMC workflow including MapRseq3 (Kalari et al., 2014) and differential expression calculated by EdgeR (McCarthy et al., 2012). Differentially expressed genes (DEGs) were filtered based on a false discovery rate (FDR) < 0.1 and an absolute FC ≥ | 1.5| called between at least one condition and the reference control sample. For RNA-seq on caerulein-treated animals, DEGs were filtered with FC ≥ | 2| and FDR < 0.05. Pathway analysis of DEGs was completed with RITAN (R package-rapid integration of annotation, network, and molecular database) (Zimmermann et al., 2019), which queries different annotation resources to analyze enrichment for an input set of genes. RITAN performs a hypergeometric test and generates FDR adjusted *p*-values, called *q*-values, for assessing pathway significance, which queries different annotation resources to analyze enrichment for an input set of genes. The Molecular Signatures Database (MSigDB) hallmark gene set collection (Liberzon et al., 2015) was used as the annotation resource. Pathways with *q*-values < 0.05 were considered enriched. Gene network and upstream regulatory analyses were performed using Ingenuity^®^ Pathway Analysis (IPA^®^; Qiagen). To quantitatively determine the percentage of infiltrating immune cell types, we utilized a “digital cytometry” method called the quanTIseq deconvolution algorithm, which estimates the absolute proportions of relevant infiltrating immune cell types from bulk RNA-seq profiles (Finotello et al., 2019). The cell type fraction scores provided by this method allow intra-sample and inter-sample comparisons of 10 immune cell type fractions. We applied the quanTIseq method through an R package called Immunedeconv (Sturm et al., 2019) on DEGs from the *Pdx1-Cre;EHMT2^*fl/fl*^*, *Pdx1-Cre;LSL-Kras*^*G*12*D*^, and *Pdx-Cre;LSL-Kras*^*G*12*D*^*;EHMT2*^*fl/fl*^ dataset (1039 DEGs), as well as the caerulein-treated animals dataset (4654 DEGs).

### Reverse Transcriptase Quantitative PCR (RT-qPCR)

Reverse transcriptase was performed using RT^2^ first strand kit (Qiagen) following manufacturer’s protocol. Real-time PCR was performed in a volume of 20 ul using SYBR Green Master Mix and CFX96 Real Time System (Bio-Rad). Primer sequences are shown in Supplementary Table 2. RT^2^ Profiler PCR Array (SA Biosciences, Qiagen) was used to examine the expression patterns of 84 genes involved in cell cycle. The array was performed following manufacturer instructions. ddCT was obtained using Qiagen software and all groups were normalized to *Pdx1-Cre* mice, with an absolute FC ≥ | 1.5| for the generation of a heatmap using R-studio.

### Senescence β-Galactosidase Staining

Senescence-associated β-galactosidase activity was assessed in fresh pancreas tissue using the Cell Signaling β-Galactosidase Staining kit, according to manufacturer’s protocol (Cell Signaling Technologies, Cat# 9860S). Briefly, under terminal anesthesia, pancreas from *LSL-Kras*^*G*12*D*^*;EHMT2*^+/+^ and *LSL-Kras*^*G*12*D*^*;EHMT2*^*fl/fl*^ with both *Pdx1-Cre and P48^*Cre/+*^* drivers were collected, washed in PBS (1×) and placed in a well of a 12-well dish containing 1 mL of fixative solution for 15 min. Pancreas were then rinsed twice with PBS (1×) and stained with β-galactosidase solution at 37°C overnight in a dry incubator. Next morning, pancreata were washed with PBS (1×), and images acquired.

### Ethics Statement

Animal care and all protocols were reviewed and approved by the Institutional Animal Care and Use Committees of Mayo Clinic Rochester (IACUC protocols A00002240-16 and A24815) and the Medical College of Wisconsin (AUA00005963).

### Data Availability

RNA-seq datasets that support the findings of this study have been deposited to the public database repository Gene Expression Omnibus (GEO) under accession code GSE169525.

## Results

### Genetic Inactivation of *EHMT2* Interrupts Kras^*G*12*D*^-Driven Initiation at the ADM Stage

**FIGURE 1:**
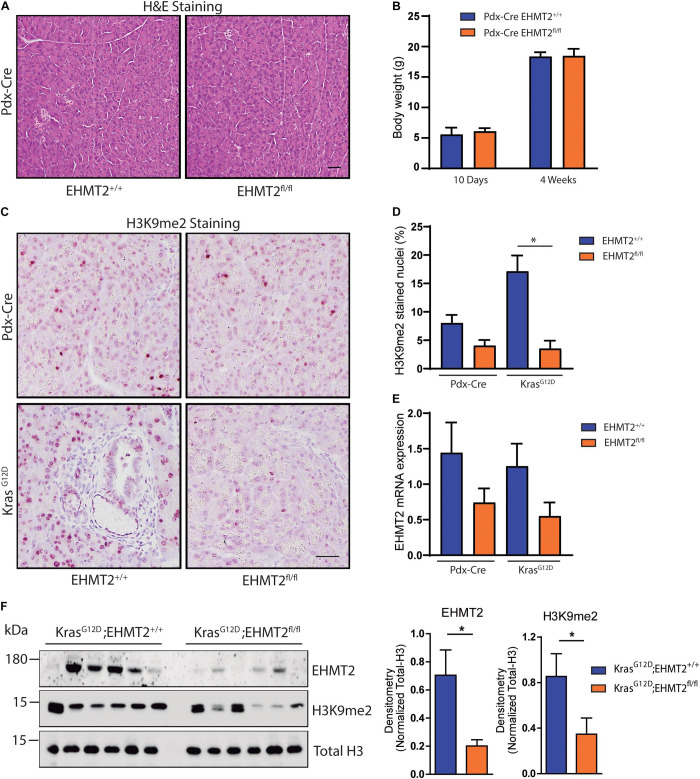
Conditional knockout of EHMT2 driven by Pdx1-Cre does not impede normal development or architecture of the mouse pancreas. **(A)** Representative images from H&E stained pancreas tissues from *Pdx1-Cre;EHMT2*^+/+^ and *Pdx1-Cre;EHMT2^*fl/fl*^* mice. No significant histologic or architectural changes were found. Scale = 50 μM. **(B)** Graph depicts body weight from *Pdx1-Cre;EHMT2*^+/+^ and *Pdx1-Cre;EHMT2^*fl/fl*^* mice at 10 days (*n* = 3 *Pdx1-Cre;EHMT2*^+/+^ and *n* = 4 *Pdx1-Cre;EHMT2^*fl/fl*^*) and 4 weeks (*n* = 16 *Pdx1-Cre;EHMT2*^+/+^ and *n* = 7 *Pdx1-Cre;EHMT2^*fl/fl*^*) of age. **(C)** Representative images of H3K9me2 IHC on pancreatic tissues from *Pdx1-Cre* and *Pdx1-Cre;LSL-Kras*^*G*12*D*^ mice with WT *EHMT2* alleles (*EHMT2*^+/+^) or crossed to homozygous floxed *EHMT2* (*EHMT2*^*fl/fl*^) animals. Scale = 50 μM. **(D)** Percentage of nuclei positive for H3K9me2 was quantified from a minimum of 5 random fields at 10× magnification (*n* = 3/group). **(E)** Levels of *EHMT2* transcript were measured by RT-PCR on RNA isolated from whole pancreas of *Pdx1-Cre;EHMT2*^+/+^ and *Pdx1-Cre;EHMT2^*fl/fl*^* mice, as well as after crossing to *LSL-Kras*^*G*12*D*^ animals (*n* = 8/group). The *EHMT2* levels were normalized to GAPDH, B2M and β-actin transcripts. **(F)** Protein lysates from whole pancreas of *Pdx1-Cre;LSL-Kras*^*G*12*D*^ and *Pdx1-Cre;LSL-Kras*^*G*12*D*^*;EHMT2*^*fl/fl*^ mice were evaluated. Left: Western blots were probed with antibodies against EHMT2 and H3K9me2. Total H3 was used as loading control. Right: Graph depicts relative densitometry values for EHMT2 and H3K9me2 levels normalized to total H3 (*n* = 6/group). * indicates *p*-value ≤ 0.05. All data is expressed as mean ± SEM.

**FIGURE 2:**
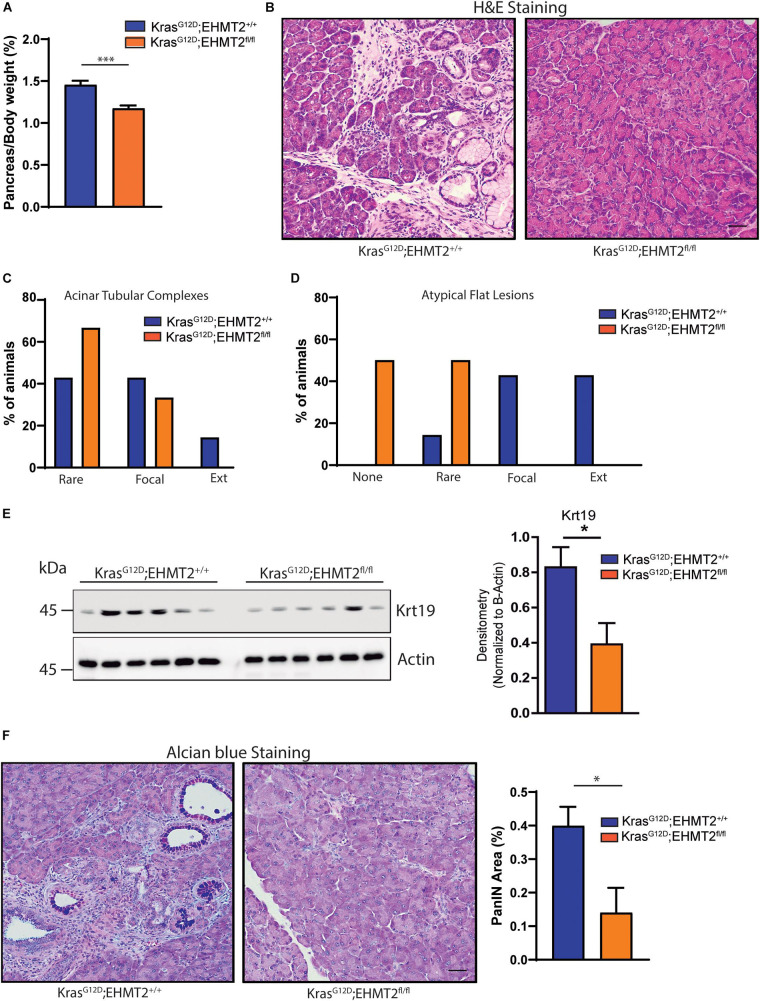
Genetic inactivation of *EHMT2* antagonizes increased pancreas-to-body weight ratios and PanIN development induced by the Kras^*G*12*D*^ mouse model. **(A)** Graph shows reduced pancreas-to-body weight ratios after *EHMT2* inactivation in *Pdx1-Cre;LSL-Kras*^*G*12*D*^ mice (*n* = 16) compared to *Pdx1-Cre;LSL-Kras^*G*12*D*^;EHMT2*^+/+^ animals (*n* = 14). **(B)** Representative H&E images of pancreatic tissue from *Pdx1-Cre;LSL-Kras^*G*12*D*^;EHMT2*^+/+^ (left) and *Pdx1-Cre;LSL-Kras*^*G*12*D*^*;EHMT2*^*fl/fl*^ (right) mice. Scale = 50 μM. Graph represents scoring from histological assessment of precursor acinar tubular complexes **(C)** and atypical flat lesions **(D)** in pancreatic tissue from *Pdx1-Cre;LSL-Kras^*G*12*D*^;EHMT2*^+/+^ (*n* = 7) and *Pdx1-Cre;LSL-Kras*^*G*12*D*^*;EHMT2*^*fl/fl*^ (*n* = 6) animals. Lesions were classified as none (for atypical flat lesions only), rare, focal or extensive (Ext). **(E)** Cytokeratin 19 (Krt19) levels were examined as a surrogate for the amount of duct-like epithelium, or ADM, in protein lysates from *Pdx1-Cre;LSL-Kras^*G*12*D*^;EHMT2*^+/+^ and *Pdx1-Cre;LSL-Kras*^*G*12*D*^*;EHMT2*^*fl/fl*^ animals. Left: Western blots were probed with an antibody against Krt19, and β-actin was used as loading control. Right: Graph depicts relative densitometry values for Krt19 levels normalized to β-actin (*n* = 6/group). **(F)** Alcian blue staining was used to quantify the burden of mucin-rich PanIN lesions. Left: Representative images from Alcian blue staining on *Pdx1-Cre;LSL-Kras^*G*12*D*^;EHMT2*^+/+^ and *Pdx1-Cre;LSL-Kras*^*G*12*D*^*;EHMT2*^*fl/fl*^ pancreas tissue. Scale = 50 μM. Left: quantification of Alcian blue-positive PanIN lesions expressed as percentage (%) of pancreas area from *Pdx1-Cre;LSL-Kras^*G*12*D*^;EHMT2*^+/+^ (*n* = 7) and *Pdx1-Cre;LSL-Kras*^*G*12*D*^*;EHMT2*^*fl/fl*^ (*n* = 6) mice. ^∗^ indicates *p*-value ≤ 0.05, and ^∗∗∗^ indicates *p*-value ≤ 0.001. All data is expressed as mean ± SEM.

By regulating H3K9me2 levels, EHMT2 plays a role in human pancreatic cancer (Yuan et al., 2012, 2013; Casciello et al., 2015; Pan et al., 2016). Initial studies in *P48*^*Cre/+*^ *Kras*^*G*12*D*^ mice indicated that EHMT2 deficiency prolonged survival by reducing PanIN growth, which was accompanied by a decreased number of phosphorylated Erk-positive and Dclk1-positive cells, both populations that contribute to PDAC initiation (Kato et al., 2020). To extend these studies, here, we use several distinct models; two purely genetic ones to express Kras^*G*12*D*^ for studying initiation combined with *EHMT2* deletion (*Pdx-Cre;LSL-Kras*^*G*12*D*^*;EHMT2*^*fl/fl*^ and *P48*^*Cre*/+^*;LSL-Kras*^*G*12*D*^*;EHMT2*^*fl/fl*^), an inducible Cre to investigate *EHMT2* inactivation *ex vivo* (*CAGGCre-ER*^TM^;*EHMT2*^*fl/fl*^), as well as the model of pancreatitis-associated promotion (Guerra et al., 2007). Prior to crossing with *Kras*^*G*12*D*^, both *Pdx1-Cre*;*EHMT2*^*fl/fl*^ and *P48^*Cre/+*^;EHMT2^*fl/fl*^* reproduced at Mendelian ratios and thrived similar to controls. Pancreata from 10-day and 4 to 6-week-old animals demonstrated normal structure and histology (Figure 1A and Supplementary Figure 1A). Body weights of *EHMT2* knockout mice were not different from controls for both *Pdx1-Cre* and *P48*^*Cre/+*^ animals (Figure 1B and Supplementary Figure 1B). The pancreatic cell nuclei from control animals were positive for H3K9me2, while from *EHMT2*^*fl/fl*^ mice displayed global reduction in H3K9me2 (8.0 ± 1.4% for *Pdx1-Cre* vs. 4.0 ± 1.0% for *Pdx1-Cre;EHMT2^*fl/fl*^*, *p* = 0.08; Figures 1C,D). Reverse transcriptase quantitative PCR (RT-qPCR) showed that *Pdx1-Cre;EHMT2^*fl/fl*^* mice display reduced *EHMT2* levels in whole pancreas (1.94 ± 0.31 FC) (Figure 1E). *EHMT2* inactivation in the *P48*^*Cre/+*^ model also showed reduced H3K9me2 by IHC (9.02 ± 1.26% for *P48*^*Cre/+*^ vs. 1.85 ± 0.28% for *P48^*Cre/+*^;EHMT2^*fl/fl*^*, *p* < 0.05; Supplementary Figures 1C,D) with a reduction in *EHMT2* transcript by RT-qPCR (3.6 ± 0.55 FC, Supplementary Figure 1E). Cre-mediated *EHMT2* exon excision in the pancreas of *Pdx1-Cre;EHMT2^*fl/fl*^* and *P48^*Cre/+*^;EHMT2^*fl/fl*^* mice was detected by PCR (Supplementary Figure 1F). Notably, for the *Pdx1-Cre* or *P48*^*Cre/+*^-driven models, organ-specific *EHMT2* inactivation occurs at embryonic day 8.5 or 9.5, respectively (Herreros-Villanueva et al., 2012). Therefore, the H3K9me2 pathway is not required for pancreas exocrine development independent of the *Cre* model used for *EHMT2* inactivation. Subsequently, we crossed *Pdx1-Cre;EHMT2^*fl/fl*^* and *P48^*Cre/+*^;EHMT2^*fl/fl*^* animals to *LSL-Kras*^*G*12*D*^ mice. IHC staining demonstrated that *LSL-Kras^*G*12*D*^;EHMT2^*fl/fl*^* mice displayed global reduction of H3K9me2 (17.1 ± 2.7% for *Pdx-Cre;LSL-Kras*^*G*12*D*^ vs. 3.5 ± 1.3% for *Pdx-Cre;LSL-Kras*^*G*12*D*^*;EHMT2*^*fl/fl*^, *p* < 0.05; Figures 1C,D). We also detected a 2.27 ± 0.25 FC reduction of *EHMT2* transcript in whole pancreas from *Pdx-Cre;LSL-Kras*^*G*12*D*^*;EHMT2*^*fl/fl*^ mice compared to *Pdx-Cre;LSL-Kras*^*G*12*D*^ animals (Figure 1E). Similar results were obtained using *P48*^*Cre/+*^ with reductions in the H3K9me2 mark (17.5 ± 1.3% for *P48^*Cre/+*^;LSL-Kras*^*G*12*D*^ vs. 4.6 ± 1.0% for *P48*^*Cre*/+^*;LSL-Kras*^*G*12*D*^*;EHMT2*^*fl/fl*^, *p* < 0.01; Supplementary Figures 1C,D) and *EHMT2* transcript (4.8 ± 0.25 FC for *P48*^*Cre*/+^*;LSL-Kras*^*G*12*D*^*;EHMT2*^*fl/fl*^ vs. *P48^*Cre/+*^;LSL-Kras*^*G*12*D*^ mice, *p* < 0.001; Supplementary Figure 1E). Western blots on pancreas protein lysates from *Pdx1-Cre;LSL-Kras*^*G*12*D*^*;EHMT2*^*fl/fl*^ animals showed reduction in EHMT2 and H3K9me2 levels when compared to *Pdx-Cre;LSL-Kras*^*G*12*D*^ (3.5 ± 0.2 FC for EHMT2, *p* < 0.05 and 2.46 ± 0.34 FC for H3K9me2, *p* < 0.05, respectively, in *Pdx1-Cre;LSL-Kras*^*G*12*D*^*;EHMT2*^*fl/fl*^ vs. *Pdx1-Cre;LSL-Kras*^*G*12*D*^ mice; Figure 1F). Noteworthy, while Kras^*G*12*D*^ expression results in larger pancreas size (Hingorani et al., 2003), knockout of *EHMT2* counteracts this effect (pancreas-to-body weight ratios: 1.45 ± 0.04% for *Pdx1-Cre;LSL-Kras*^*G*12*D*^ vs. 1.17 ± 0.03% for *Pdx1-Cre;LSL-Kras*^*G*12*D*^*;EHMT2*^*fl/fl*^ mice, *p* < 0.001; Figure 2A). Similar results were found in *P48^*Cre/+*^;LSL-Kras*^*G*12*D*^ mice (1.43 ± 0.06% from *P48^*Cre/+*^;LSL-Kras*^*G*12*D*^ vs. 1.1 ± 0.02% from *P48*^*Cre*/+^*;LSL-Kras*^*G*12*D*^*;EHMT2*^*fl/fl*^ mice, *p* < 0.001; Supplementary Figure 2A). Hence, while genetic inactivation of *EHMT2* is tolerated by the organ during development, its presence appears to be critical for supporting Kras^*G*12*D*^-induced pancreatic neoplastic growth (Figure 2B and Supplementary Figure 2B). Next, blinded histological examination of tissues was performed by two separate pathologists (V.A. and B.P.). We found that Kras^*G*12*D*^-expressing *EHMT2* knockout mice with both Cre models had limited acinar tubular complexes and atypical flat lesions with a reduction in ADM formation (Figures 2C,D and Supplementary Figures 2C,D), which is the earliest morphological hallmark of PDAC initiation (Esposito et al., 2014; Murtaugh and Keefe, 2015). In support of this observation, western blot analyses of lysates from *Kras*^*G*12*D*^-expressing pancreata showed reduced cytokeratin 19 (Krt19) levels in *Pdx-Cre;LSL-Kras*^*G*12*D*^*;EHMT2*^*fl/fl*^ animals (2.12 ± 0.28 FC on *Pdx-Cre;LSL-Kras*^*G*12*D*^*;EHMT2*^*fl/fl*^ vs. *Pdx-Cre;LSL-Kras*^*G*12*D*^ mice, Figure 2E). Figure 2F demonstrates that *Pdx1-Cre;LSL-Kras*^*G*12*D*^*;EHMT2*^*fl/fl*^ mice indeed had reduced PanIN when compared to *Pdx1-Cre;LSL-Kras*^*G*12*D*^ mice, (0.14 ± 0.07% vs. 0.39 ± 0.05%, respectively; *p* < 0.05). Similar findings were obtained with *P48^*Cre/+*^;LSL-Kras*^*G*12*D*^ and *P48*^*Cre*/+^*;LSL-Kras*^*G*12*D*^*;EHMT2*^*fl/fl*^ animals (0.56 ± 0.05% vs. 0.23 ± 0.08%, respectively; *p* < 0.05, Supplementary Figure 2E). Our studies, stringently obtained with the two most often used Cre-drivers for KRAS-mediated initiation, suggest that EHMT2 inactivation antagonizes PanIN formation, as early as its ADM precursor stage.

**FIGURE 3:**
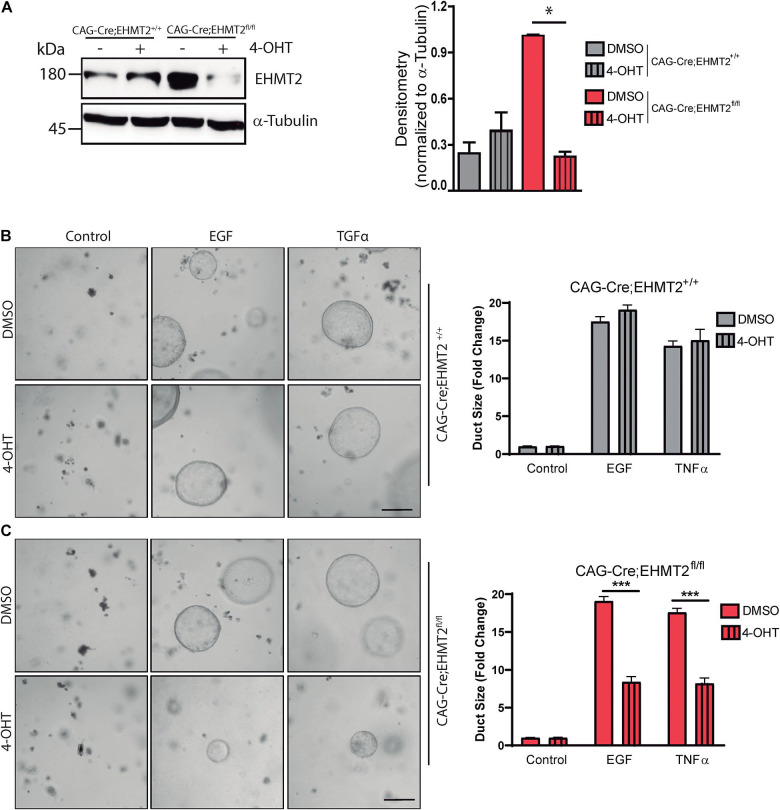
EHMT2 inactivation impairs EGFR-KRAS-MAPK pathway-driven ADM in primary acinar cell explant cultures. **(A)** Acinar cell explant cultures derived from *CAGGCre-ER^TM^;EHMT2*^+/+^ and *CAGGCre-ER^TM^;EHMT2^*fl/fl*^* animals were grown in the absence (–) or presence (+) of 4-Hydroxytamoxifen (4-OHT) to achieve *EHMT2* knockout. Left: Western blot evaluation of protein lysates from acinar explant cultures was performed to confirm that 4-OHT treatment reduced EHMT2 levels in cells derived from *CAGGCre-ER^TM^;EHMT2^*fl/fl*^* mice. Alpha-tubulin was used as a loading control. Right: Graph depicts relative densitometry values for EHMT2 normalized to tubulin. Representative phase contrast images of *CAGGCre-ER^TM^;EHMT2*^+/+^ **(B)** and *CAGGCre-ER^TM^;EHMT2^*fl/fl*^* **(C)** explant cultures after 5–6 days of EGF (20 ng/mL) or TGFα (50 ng/mL) exposure to induce ADM. Cells were also treated with vehicle (DMSO) or 4-OHT to inactivate *EHMT2*. Right: Quantification of duct-like structures is shown as fold-change in size relative to control. * indicates *p*-value ≤ 0.05, and *** indicates *p*-value ≤ 0.001. All data is expressed as mean ± SEM (experiment performed in triplicate).

To mechanistically complement our *in vivo* data, we next investigated whether EHMT2 is involved in the ADM process, using a genetically engineered *ex vivo* approach with acinar explants from a tamoxifen-inducible model of *EHMT2* knockout. This model carries a *CAGGCre-ER*^TM^ transgene, which has the chicken β-actin promoter/enhancer coupled with the cytomegalovirus immediate-early enhancer to drive high levels of Cre expression, allowing this enzyme to translocate into the nucleus (Hayashi and McMahon, 2002) upon tamoxifen treatment. Acinar cells from *CAGGCre-ER*^TM^ and *CAGGCre-ER^TM^;EHMT2^*fl/fl*^* animals were isolated and grown in 3D matrigel in the absence (−) or presence (+) of 4-Hydroxytamoxifen (4-OHT) to inactivate *EHMT2*. Western blot showed that EHMT2 protein levels were reduced after 4-OHT treatment (Figure 3A). Subsequently, acinar cell explants were cultured in presence of EGF or TGFα for 5–6 days to activate the KRAS-MAPK pathway, which induces *ex vivo* ADM formation. Phase microscopy of 3D cultures showed that EGF and TGF-α induced duct-like structures from acini isolated from *CAGGCre-ER*^TM^ (−/+4-OHT) controls, demonstrating ADM formation (Figure 3B). No significant difference was observed in the size of duct-like structures formed from acini of *CAGGCre-ER*^TM^ alone animals either in the absence or presence of 4-OHT upon EGF treatment (17.51 ± 0.6 FC with DMSO vs. 19.07 ± 0.6 FC with 4-OHT) or after TGFα treatment (14.3 ± 0.7 FC with DMSO vs. 15.05 ± 1.4 FC with 4-OHT; Figure 3B). *CAGGCre-ER^TM^;EHMT2^*fl/fl*^* without 4-OHT treatment also demonstrated ADM formation after EGF and TGFα treatment (Figure 3C). However, upon EHMT2 inactivation (+4-OHT), the quantification of duct-like structures revealed significantly reduced ADM formation with EGF (19.08 ± 0.7 FC with DMSO vs. 8.36 ± 0.6 FC with 4-OHT; *p* < 0.001) or TGFα (17.58 ± 0.5 FC with DMSO vs. 8.19 ± 0.75 FC with 4-OHT; *p* < 0.001; Figure 3C). Thus, congruent with the antagonism to Kras^*G*12*D*^-mediated ADM and PanIN initiation found in *LSL-Kras^*G*12*D*^;EHMT2^*fl/fl*^* mice, loss of EHMT2 impairs the phenotypic conversion of acinar cells to more duct-like structures in 3D *ex vivo* cultures stimulated with growth factors that activate the EGF-KRAS pathway, further supporting the rigor of our *in vivo* observations.

### Kras^*G*12*D*^ Induces Heterotrimerization of the EHMT2-EHMT1-WIZ Complex, Increasing H3K9me2 Levels

**FIGURE 4:**
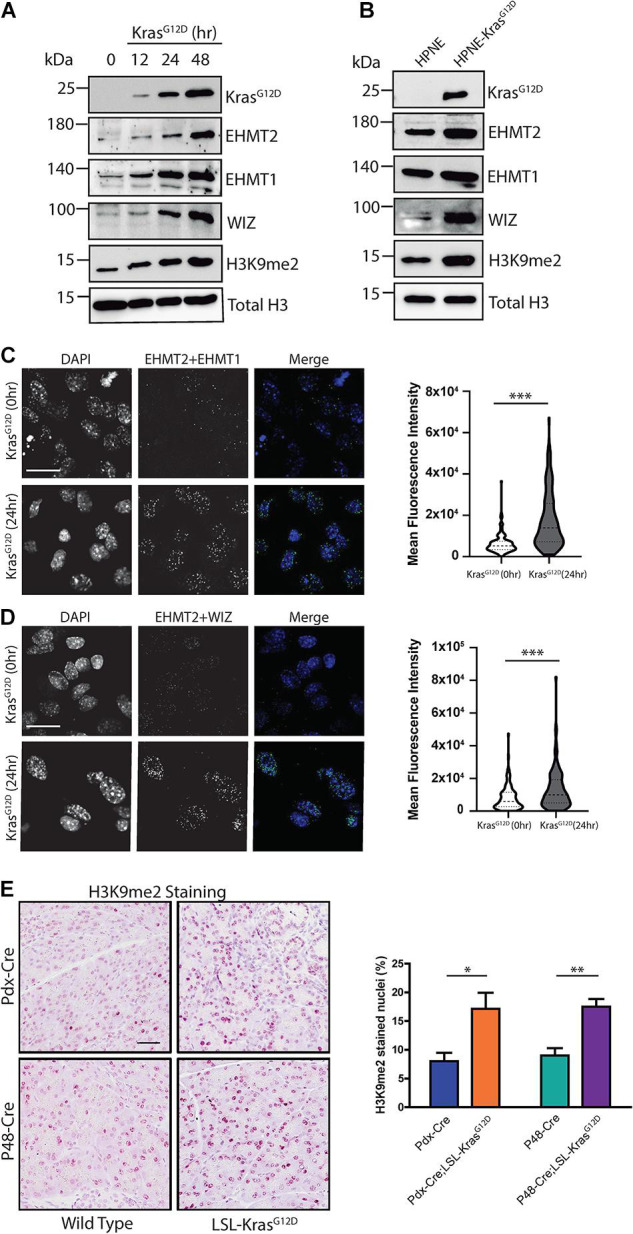
Kras^*G*12*D*^ promotes EHMT2-EHMT1-WIZ complex formation and H3K9me2 deposition. **(A)** Western blot evaluation of iKras 4292 cells after expression of Kras^*G*12*D*^. Kras^*G*12*D*^ expression was stimulated with doxycycline for 12, 24, and 48 h, then members of the EHMT2 complex, including EHMT2, EHMT1, and WIZ, as well as its product, H3K9me2, were evaluated. Induction of Kras^*G*12*D*^ was verified, and total H3 was used as loading control. Quantification is represented in Supplementary Figure 3A (experiment performed in triplicate). **(B)** Expression of KRAS^*G*12*D*^, EHMT2, EHMT1, WIZ, and H3K9me2 was evaluated by western blot in HPNE and HPNE-KRAS^*G*12*D*^ cells, developed from human pancreatic ducts. Kras^*G*12*D*^ expression is shown to be specific to HPNE-KRAS^*G*12*D*^ cells. Total H3 was used as loading control. Quantification is represented in Supplementary Figure 3F (experiment performed in triplicate). **(C)** Interaction between EHMT2 and EHMT1 was evaluated using PLA. Left: Representative pictures of iKras 4292 cells at 0 and 24 h after Kras^*G*12*D*^ activation. Scale = 50 μM. Right: Violin plot representing Mean Fluorescence Intensity (MFI) of EHMT2/EHMT1 positive PLA signal (experiment performed in triplicate). **(D)** Interaction between EHMT2 and WIZ was also evaluated using PLA. Left: Representative pictures of iKras 4292 cells at 0 and 24 h post-Kras^*G*12*D*^ induction. Scale = 50 μM. Right: Violin plot representing MFI of EHMT2/WIZ PLA signal (experiment performed in triplicate). **(E)** Levels of the EHMT2 product, H3K9me2, as measured by IHC staining. Left: Representative images of pancreas tissue from *Pdx1-Cre* and *P48*^*Cre/+*^ WT and Kras^*G*12*D*^-expressing mice stained for H3K9me2. Scale = 50 μM. Right: Percentage of nuclei positive for H3K9me2 was quantified from a minimum of 5 random fields at 10× magnification (*n* = 3/group). ^∗^ indicates *p*-value ≤ 0.05, ^∗∗^ indicates *p*-value ≤ 0.01, and ^∗∗∗^ indicates *p*-value ≤ 0.001. All data is expressed as mean ± SEM.

We also investigated whether Kras^*G*12*D*^ behaves as an upstream regulator of EHMT2 activity. For this purpose, we utilized a GEMM-derived PDAC cell model in which oncogenic *Kras*^*G*12*D*^ expression is induced by doxycycline (Collins et al., 2012) (iKras 4292) to evaluate downstream effects on the EHMT2-H3K9me2 epigenetic pathway. We induced *Kras*^*G*12*D*^ expression and collected lysates at various timepoints to examine levels of proteins from this pathway (Figure 4A). Kras^*G*12*D*^ protein levels were detected at 12 h, reaching maximum after 48 h (10.57 ± 1.6-fold change (FC) vs. 0 h; *p* < 0.01; Supplementary Figure 3A). We found that Kras^*G*12*D*^ increased protein levels for both, H3K9 methyltransferases EHMT2 and EHMT1, peaking at 48 h (4.6 ± 1.2 FC for EHMT2 at 48 h vs. 0 h, *p* < 0.05, and 4.18 ± 1.7 FC for EHMT1 at 48 h compared to 0 h, *p* < 0.01; Supplementary Figure 3A). EHMT2 and EHMT1 form an enzymatically active heterotrimer with WIZ, which stabilizes the complex on chromatin (Simon et al., 2015). We also observed increased levels of WIZ (3.2 ± 1.3 FC at 48 h vs. 0 h; *p* < 0.05; Supplementary Figure 3A). Levels of the H3K9me2 mark increased upon Kras^*G*12*D*^ induction as well (2.50 ± 0.4 FC at 48 h vs. 0 h; *p* < 0.001; Supplementary Figure 3A). In addition, using immunofluorescence-based confocal microscopy, we detected increased protein levels for EHMT2, EHMT1, WIZ, and H3K9me2 (Supplementary Figures 3B–E). Indeed, the mean fluorescence intensity (MFI) for EHMT2 at baseline (0 h) was 2.81e4 ± 2.41e3 compared with 6.97e4 ± 3.21e3 at 48 h (*p* < 0.001; Supplementary Figure 3C). For EHMT1, MFI values were 2.40e4 ± 2.21e3 at 0 h vs. 6.42e4 ± 3.91e3 at 48 h (*p* < 0.001; Supplementary Figure 3C). For WIZ, the MFI increased from 3.59e4 ± 3.25e3 at 0 h to 8.60e4 ± 4.96e3 at 48 h (*p* < 0.001; Supplementary Figure 3D). H3K9me2 MFI values were 1.38e4 ± 3.51e3 for 0 h vs. 1.20e5 ± 1.01e4 at 48 h (*p* < 0.001; Supplementary Figure 3E). Lastly, using a set of genetically engineered human pancreatic duct-derived cell models specifically designed to study KRAS^*G*12*D*^, hTERT-HPNE E6/E7 (HPNE), and hTERT-HPNE E6/E7 KRAS^*G*12*D*^ (HPNE-KRAS^*G*12*D*^), we observed a similar increase in the EHMT2-EHMT1-WIZ complex along with di-methylation of its H3K9 substrate. Congruent with the data described above, HPNE-KRAS^*G*12*D*^ cells displayed higher protein levels of EHMT2, EHMT1, WIZ, and H3K9me2 compared to its HPNE counterpart (Figure 4B and Supplementary Figure 3F). Thus, activation of oncogenic KRAS, the earliest genetic event in PDAC, results in increased levels of the EHMT2 complex and its catalytic product, the H3K9me2 mark.

Next, since stability and methyltransferase activity depends on formation of the complex (Ueda et al., 2006), we immunopurified EHMT2 from iKras 4292 cells and performed mass spectrometry, which revealed increased hetero-trimerization of EHMT2, EHMT1, and WIZ after 24 h of Kras^*G*12*D*^ expression (Supplementary Figure 3G). We also used PLA to detect, *in situ*, the interaction between endogenous EHMT2 and EHMT1 or WIZ (Figures 4C,D and Supplementary Figures 4A,B). PLA signals from EHMT2 + EHMT1 complexes increased after Kras^*G*12*D*^ induction, with MFI of 6.29e3 ± 3.98e2 at baseline and 1.75e4 ± 1.12e3 after 24 h (*p* < 0.001, Figure 4C). This effect was also accompanied by increased PLA signals for EHMT2 + WIZ complexes upon Kras^*G*12*D*^ expression (MFI of 7.89e3 ± 6.47e2 at baseline vs. 1.33e4 ± 1.16e3 at 24 h, *p* < 0.001, Figure 4D). The induction of EHMT2, EHMT1, and WIZ protein levels, as well as the formation of their complex detected by mass spectrometry and PLA are important since we did not detect changes in their mRNA levels either in the absence or presence of Kras^*G*12*D*^ (Supplementary Figure 4C). This result was recapitulated in the human KRAS^*G*12*D*^ cell models, as we found no statistical difference in *EHMT2*, *EHMT1* and *WIZ* transcript levels from HPNE cells compared to HPNE-KRAS^*G*12*D*^ cells (Supplementary Figure 4D). These comparable transcript levels in the absence and presence of oncogene activation suggest that KRAS^*G*12*D*^-effects on the EHMT2 complex are possibly not via transcriptional regulation of these genes but rather potentially due to protein stability. Given this result, we also performed staining for the H3K9me2 mark, by immunohistochemistry (IHC) in wild-type and Kras^*G*12*D*^ expressing mice to translate these effects to the *in vivo* situation. Pancreata were harvested from both, *Pdx1-Cre;LSL-Kras*^*G*12*D*^ and *P48^*Cre/+*^;LSL-Kras*^*G*12*D*^ mouse models. Upon quantification of positively stained nuclei compared to total nuclei, we found that *Pdx1-Cre;LSL-Kras*^*G*12*D*^ animals had higher levels of H3K9me2 compared to control *Pdx1-Cre* mice (17.1 ± 2.7% vs. 8.0 ± 1.4%, *p* < 0.05; Figure 4E). Similar results were obtained when Kras^*G*12*D*^ activation was driven by Cre expression from the *Ptf1a-P48* promoter via knock-in (17.5 ± 1.3% in *P48^*Cre/+*^;LSL-Kras*^*G*12*D*^ mice compared to 9.0 ± 2.1% in *P48*^*Cre/+*^ controls, *p* < 0.01; Figure 4E). Thus, based on these collective data, we conclude that EHMT2, EHMT1 and WIZ proteins form a stable complex upon Kras^*G*12*D*^ activation, which leads to enhanced deposition of the H3K9me2 mark, supporting a role for this epigenetic pathway downstream of the most commonly mutated oncogene in PDAC.

### EHMT2 Inactivation Establishes a Transcriptional Landscape Antagonistic to PDAC Initiation

**FIGURE 5:**
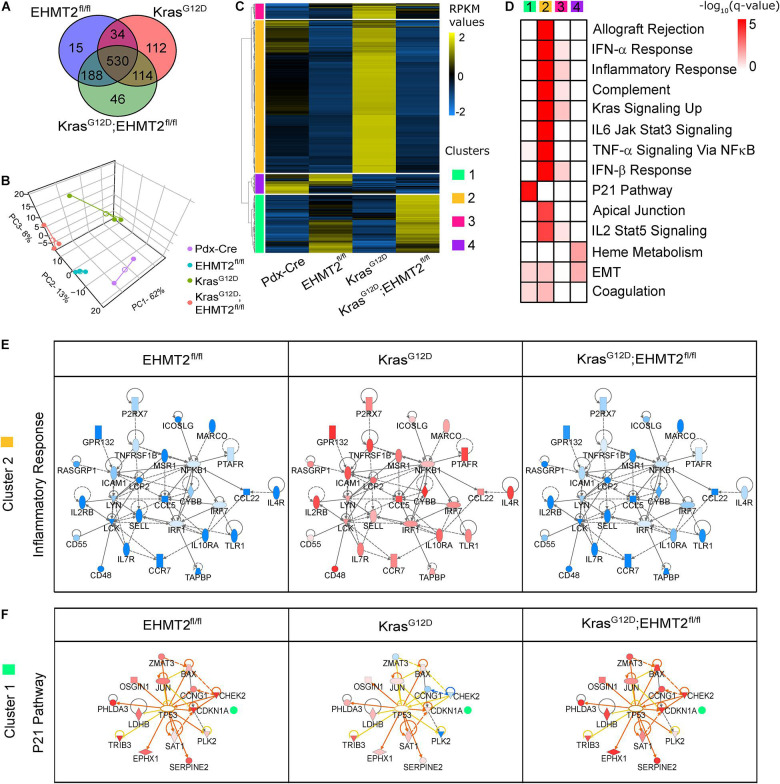
RNA-seq analysis of conditional knockout of EHMT2 in Kras^*G*12*D*^-expressing animals reveals an antagonistic transcriptional response. **(A)** Venn diagram of differentially expressed genes (DEGs) from *Pdx1-Cre;EHMT2^*fl/fl*^* (*n* = 3), *Pdx1-Cre;LSL-Kras^*G*12*D*^;EHMT2*^+/+^ (*n* = 3), and *Pdx1-Cre;LSL-Kras*^*G*12*D*^*;EHMT2*^*fl/fl*^ (*n* = 3) mice relative to *Pdx1-Cre* control animals (*n* = 2) by RNA-seq. **(B)** PCA plot comparing genotype groups with centroids (open circles) and individual animals (filled circles) using RPKM expression values of DEGs. **(C)** Heatmap depicts RPKM expression levels of DEGs normalized to the z-scale. Hierarchical clustering representing four major expression patterns is labeled on the left of the heatmap (green, Cluster 1; orange, Cluster 2; pink, Cluster 3; and purple, Cluster 4). Average of normalized RPKM values is represented in yellow (up), blue (down), and black (no change). **(D)** Pathway enrichment analysis of gene clusters in panel **(C)**. Color scale denotes –log_10_(*q*-value) for significance of each represented pathway. **(E)** The inflammatory gene network enriched in Cluster 2, which represents genes upregulated by *Kras*^*G*12*D*^ activation but downregulated in animals carrying the *EHMT2*^*fl/fl*^ allele. Gene fold changes with respect to controls are represented with red (up) and blue (down) nodes. **(F)** Upstream regulatory analysis of genes in Cluster 1, which represent the P21 pathway upregulated in *EHMT2*^*fl/fl*^ mice. Color scheme of nodes is same as in panel **(E)**. Outlined central node represents the predicted link with the upstream regulator, TP53, while orange lines and blue lines indicate predicted relationships that lead to activation or inhibition, respectively. Yellow lines designate that the predicted relationship is inconsistent with gene expression, and gray lines denote no predicted effect.

Oncogenic KRAS mounts transcriptional responses that program the growth-promoting phenotype that characterizes pancreatic cancer initiation (Waters and Der, 2018). This observation, along with the fact that EHMT2 is a well-known regulator of transcriptional responses (Shankar et al., 2013), led us to carry out RNA-seq from whole pancreas to evaluate gene expression networks affected by *EHMT2* inactivation. We performed these experiments comparatively in four murine lines, namely *Pdx1-Cre*, *Pdx1-Cre;EHMT2^*fl/fl*^*, *Pdx1-Cre;LSL-Kras*^*G*12*D*^, and *Pdx-Cre;LSL-Kras*^*G*12*D*^*;EHMT2*^*fl/fl*^. We found a total of 767 DEGs (with 257 upregulated and 510 downregulated) in *Pdx1-Cre;EHMT2^*fl/fl*^*, 790 DEGs (691 up, 99 down) in *Pdx1-Cre;LSL-Kras*^*G*12*D*^ and 878 DEGs (305 up, 573 down) in *Pdx1-Cre;LSL-Kras*^*G*12*D*^*;EHMT2*^*fl/fl*^, normalized to *Pdx1-Cre* mice, with an absolute FC ≥ | 1.5| and FDR < 0.1 (Figure 5A and Supplementary Table 3). The largest subset of regulated genes across all three experimental conditions was comprised of 530 DEGs. The second largest subset of 188 DEGs were differentially expressed only in *EHMT2* inactivated samples, irrespective of *Kras*^*G*12*D*^ (Figure 5A). On the other hand, *Kras*^*G*12*D*^ carrying mice showed 114 DEGs irrespective of their *EHMT2* status (Figure 5A). Principal component analysis (PCA) using all 1039 DEGs led to separation of samples by cluster and experimental conditions (Figure 5B). Next, we performed calculations of distances among cluster centroids for experimental samples and controls (marked by empty circles in Figure 5B), as a similarity measure among genetic conditions. The distance between the *EHMT2* inactivated groups was 21.7 while the distance between *Pdx1-Cre;EHMT2^*fl/fl*^* and *Pdx1-Cre;LSL-Kras*^*G*12*D*^ was 46.8 and between the two *Kras*^*G*12*D*^ activated groups was 48.7. Thus, *EHMT2* inactivation in the *Pdx1-Cre;LSL-Kras^*G*12*D*^ EHMT2^*fl/fl*^* animals clustered much closer to *Pdx1-Cre;EHMT2^*fl/fl*^* and significantly separated from *Kras*^*G*12*D*^ mice with *EHMT2* intact, indicating that *EHMT2* inactivation is characterized by a transcriptional profile that is functionally antagonistic to oncogenic KRAS. Hierarchical clustering of normalized DEG across all three conditions (1039 genes) revealed four major expression patterns (Figure 5C). When compared to *Pdx1-Cre* controls and *Kras*^*G*12*D*^ animals, the 245 genes defining cluster 1 (green) were upregulated in *EHMT2*^*fl/fl*^ and *Kras*^*G*12*D*^*;EHMT2*^*fl/fl*^ mice, while 647 genes characterized cluster 2 (yellow) with genes downregulated upon *EHMT2* inactivation, either alone or with *Kras*^*G*12*D*^. Cluster 3 (pink) displayed 65 genes upregulated in *Kras*^*G*12*D*^ animals, and Cluster 4 (purple) was defined by 82 genes downregulated in *Kras*^*G*12*D*^ animals, independent of *EHMT2* status. Notably, ADM and PanIN-related mRNA markers, such as *Gkn1*, *Gkn2*, and *Muc5a*, were downregulated in *Pdx1-Cre;LSL-Kras*^*G*12*D*^*;EHMT2*^*fl/fl*^ animals (Supplementary Figure 5A), supporting our histological observations. Together, these studies revealed that mice with *EHMT2* inactivated in their pancreas epithelial cells displayed a gene expression pattern antagonistic to the Kras^*G*12*D*^-regulated gene expression program.

### Growth-Inhibitory Gene Networks Are a Hallmark of EHMT2 Inactivation During KRAS-Mediated Initiation

We used different tools for data mining the biological and mechanistic significance of the gene expression networks identified by RNA-seq. Hierarchical clustering identified four main patterns of gene expression. Cluster 1 genes represented those upregulated upon *EHMT2* inactivation that remained elevated in *EHMT2*^*fl/fl*^ crossed to *Kras*^*G*12*D*^ mice. Notably, cluster 1 included a gene network corresponding to the P21 pathway (*q*-value = 1.81 × 10^–5^; Figure 5D and Supplementary Table 4), which causes growth arrest. Cluster 2 genes, downregulated by *EHMT2* inactivation, involved gene networks that participate in KRAS signaling (*q*-value = 1.5 × 10^–10^) and immunoregulatory/inflammation-related pathways (*q*-value = 2.3 × 10^–14^), (e.g., type I interferon response, IL6-Jak-Stat3 signaling, and IL2-Stat5 signaling; Supplementary Table 4). This is important since inflammatory responses are required for *Kras*^*G*12*D*^-mediated initiation (di Magliano and Logsdon, 2013). No biological pathway was found to be significant in cluster 3, while cluster 4 contained genes downregulated in *Kras*^*G*12*D*^ animals, which participate in EMT (*q*-value = 2.3 × 10^–2^). Additional gene network enrichment and visualization analyses using semantic-based algorithms (Krämer et al., 2014) showed that *EHMT2* inactivation results in antagonism of transcriptional networks corresponding to inflammatory responses as part of gene networks that participate in KRAS signaling (Figure 5E). Upstream Regulatory Analysis linked the upregulation of *Cdkn1a/p21*, *Chek2*, *Bax*, *Jun*, and *Ccng1*, among other genes, to a P53-like transcriptional network activated in *EHMT2*^*fl/fl*^ mice, and this molecular phenotype was transferred to the *Kras*^*G*12*D*^*;EHMT2*^*fl/fl*^ mice (Figure 5F). Therefore, *EHMT2* inactivation antagonizes oncogenic *Kras*^*G*12*D*^ at the molecular level, at least in part, by upregulating well-known pathways for cell cycle inhibitory checkpoints, though also unexpectedly, downregulating inflammatory responses.

RNA-seq counts from pancreas are dominated by the high expression of multiple key digestive enzyme genes (Hoang et al., 2016), occasionally masking genes expressed at lower levels that can be causal of phenotypes. For this reason, we also utilized more sensitive, pathway-specific RT-qPCR-based gene expression profiling focused on cell cycle-related transcripts to monitor the status of the entire gene expression network that regulates cell cycle. Results from *EHMT2*^*fl/fl*^, *Kras*^*G*12*D*^, or *Kras*^*G*12*D*^*;EHMT2*^*fl/fl*^ mice were normalized to those from *Pdx1-Cre* mice. Indeed, this approach revealed that *Kras*^*G*12*D*^*;EHMT2*^*fl/fl*^ and *EHMT2*^*fl/fl*^ animals clustered closer to each other than with *Kras*^*G*12*D*^, sharing a similar expression profile for cell cycle-related genes (Supplementary Figure 5B). Genes forming Cluster 1 were involved in cell cycle arrest and senescence after replication stress (*Cdkn1a/p21* and *Chek2*) (Aliouat-Denis et al., 2005; Cook, 2009), remaining upregulated in both *EHMT2*^*fl/fl*^ and *Kras*^*G*12*D*^*;EHMT2*^*fl/fl*^ mice (orange, Supplementary Figure 5B). Genes within cluster 2 contained networks that also function in replication stress responses (*Atr*, *Wee1*, *Brca1*, and *Rad9a*), which often also result in senescence induction (Gralewska et al., 2020), as well as genes, including *Skp2* and *Cdc6*, involved in origin licensing during DNA replication (Cook, 2009). These genes ranged in levels from unchanged to slightly increased by *Kras*^*G*12*D*^ but underwent further upregulation upon *EHMT2* inactivation, whether in the absence or presence of *Kras*^*G*12*D*^ (purple, Supplementary Figure 5B). Cluster 3 genes were characterized by the presence of the replication stress kinase *Chek1* and several cyclins (*Ccna2*, *Ccnb1*, *Ccnd1*, and *Ccnd2*). These genes were downregulated by *Kras*^*G*12*D*^ alone but markedly upregulated in both *EHMT2*^*fl/fl*^ and *Kras*^*G*12*D*^*;EHMT2*^*fl/fl*^ mice (green, Supplementary Figure 5B). Thus, combined these data suggest that *EHMT2* inactivation alone increases the levels of cell cycle regulatory molecules known to function as checkpoints to arrest cell growth in response to replication stress, congruent with emerging data suggesting that this protein localizes and works at the replication fork (Estève et al., 2006; Dungrawala et al., 2015; Ferry et al., 2017) and pharmacological data that has implicated a role of this protein in senescence without much mechanistic insight (Yuan et al., 2012). Notably, these expression networks were inherited when *EHMT2*^*fl/fl*^ mice were crossed to the *Kras*^*G*12*D*^ background. While cluster 4 was primarily formed by *Kras*^*G*12*D*^-downregulated transcripts, its pattern displayed a mixed profile of downregulated, unchanged, and minimally upregulated transcripts in the *EHMT2*^*fl/fl*^ and *Kras*^*G*12*D*^*;EHMT2*^*fl/fl*^ mice (red, Supplementary Figure 5B). *EHMT2*^*fl/fl*^ and *Kras*^*G*12*D*^*;EHMT2*^*fl/fl*^ mice blunted the *Kras*^*G*12*D*^-mediated downregulation of transcripts in this cluster, which encompassed DNA damage and apoptosis-related genes. Thus, these data further support the significant role that EHMT2 inactivation plays in the regulation of gene expression networks that are primarily involved in signaling for replication stress, cell cycle arrest and senescence.

### Inactivation of *EHMT2* Gives Rise to a P21-Mediated Senescent Phenotype

**FIGURE 6:**
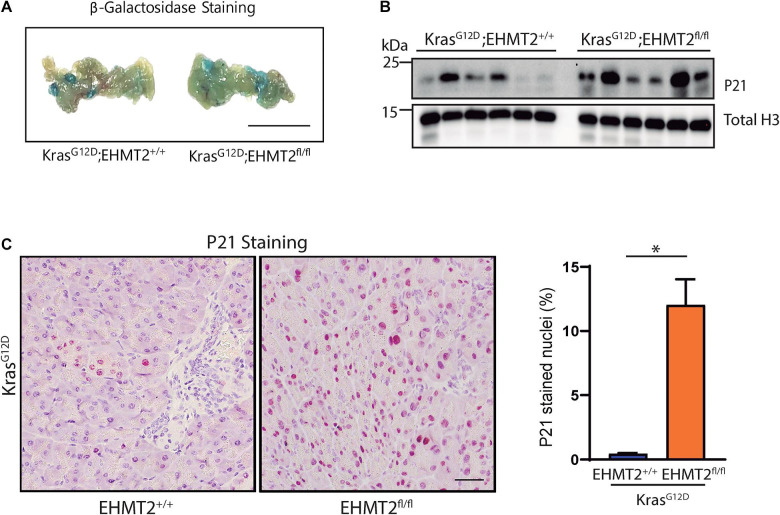
EHMT2 inactivation induces cell senescence in the Kras^*G*12*D*^-expressing mouse pancreas coincident with increased P21. **(A)** Senescence-associated β-galactosidase staining on fresh pancreas tissue extracted from *Pdx1-Cre;LSL-Kras^*G*12*D*^;EHMT2*^+/+^ and *Pdx1-Cre;LSL-Kras*^*G*12*D*^*;EHMT2*^*fl/fl*^ animals. The presence of blue staining indicates senescence. Scale = 1 mm. **(B)** Western blot evaluation of P21 protein levels in whole pancreas lysates from *Pdx1-Cre;LSL-Kras^*G*12*D*^;EHMT2*^+/+^ and *Pdx1-Cre;LSL-Kras*^*G*12*D*^*;EHMT2*^*fl/fl*^ mice. Total H3 was used as loading control (*n* = 6/group). Quantification is represented in Supplementary Figure 6B. **(C)** Left: Representative images from IHC staining for P21 on *Pdx1-Cre;LSL-Kras^*G*12*D*^;EHMT2*^+/+^ and *Pdx1-Cre;LSL-Kras*^*G*12*D*^*;EHMT2*^*fl/fl*^ pancreas tissue. Scale = 50 μM. Right: Graph represents percentage of nuclei positive for P21 as quantified from a minimum of 5 random fields at 10× magnification (*n* = 3/group). ^∗^ indicates *p*-value ≤ 0.05; Student’s *t*-test with Welch’s correction. All data is expressed as mean ± SEM.

Based on the discovery that *EHMT2* inactivation upregulates the *Cdkn1a/p21-Chek2* pathway, which when participating in prolonged cell cycle arrest, leads to senescence (Aliouat-Denis et al., 2005; d’Adda di Fagagna, 2008), we hypothesized that loss of EHMT2 may prevent Kras^*G*12*D*^-mediated cell growth, at least in part, through this mechanism. Thus, we performed fresh tissue senescence-associated β-galactosidase staining on *Pdx1-Cre;LSL-Kras*^*G*12*D*^ and *Pdx1-Cre;LSL-Kras*^*G*12*D*^*;EHMT2*^*fl/fl*^ pancreas tissue. Tissue from *Kras*^*G*12*D*^*;EHMT2*^*fl/fl*^ mice displayed marked blue staining indicating increased senescence compared to *Kras*^*G*12*D*^ mice with *EHMT2* intact (Figure 6A). Similar results were obtained with *P48*^*Cre*/+^*;Kras*^*G*12*D*^*;EHMT2*^*fl/fl*^ (Supplementary Figure 6A). Concomitantly, using western blot using lysates from *Pdx1-Cre;LSL-Kras*^*G*12*D*^ and *Pdx1-Cre;LSL-Kras*^*G*12*D*^*;EHMT2*^*fl/fl*^ mice (*n* = 6/group), we found that *EHMT2* inactivation, which upregulated *Cdkn1a/p21* mRNA, also increased the levels of its encoded cell cycle regulator protein (2.19 ± 0.38 FC for *Pdx1-Cre;LSL-Kras*^*G*12*D*^*;EHMT2*^*fl/fl*^ vs. *Pdx1-Cre;LSL-Kras*^*G*12*D*^; *p* < 0.05; Figure 6B and Supplementary Figure 6B). IHC performed on tissue from these animals further supported the increase of P21 protein in the nuclei of pancreas cells with EHMT2 inactivated (0.45 ± 0.05% from *Pdx1-Cre;LSL-Kras*^*G*12*D*^ vs. 12.03 ± 2.00% from *Pdx1-Cre;LSL-Kras*^*G*12*D*^*;EHMT2*^*fl/fl*^ animals, *p* < 0.05; and 0.26 ± 0.05% from *P48^*Cre/+*^;LSL-Kras*^*G*12*D*^ vs. 4.35 ± 0.06% from *P48*^*Cre*/+^*;LSL-Kras*^*G*12*D*^*;EHMT2*^*fl/fl*^ mice, *p* < 0.001; Figure 6C and Supplementary Figure 6C). Thus, *EHMT2* inactivation exerts its inhibitory effects on Kras^*G*12*D*^, at least in part, by one cellular mechanism, namely senescence. At the molecular level, we reveal that genetic inactivation of *EHMT2* generates a transcriptional profile, which is antagonistic to oncogenesis and transferred to the cross with *Kras*^*G*12*D*^. This profile is represented by linked nodes that converge on the upregulation of P21-mediated pathways, a finding that is validated by evidence gathered at the transcriptional level, protein level, and by *in situ* immunostaining.

### *EHMT2* Inactivation Modifies the Immune Landscape Underlying Kras^*G*12*D*^-Mediated PDAC Initiation

**FIGURE 7:**
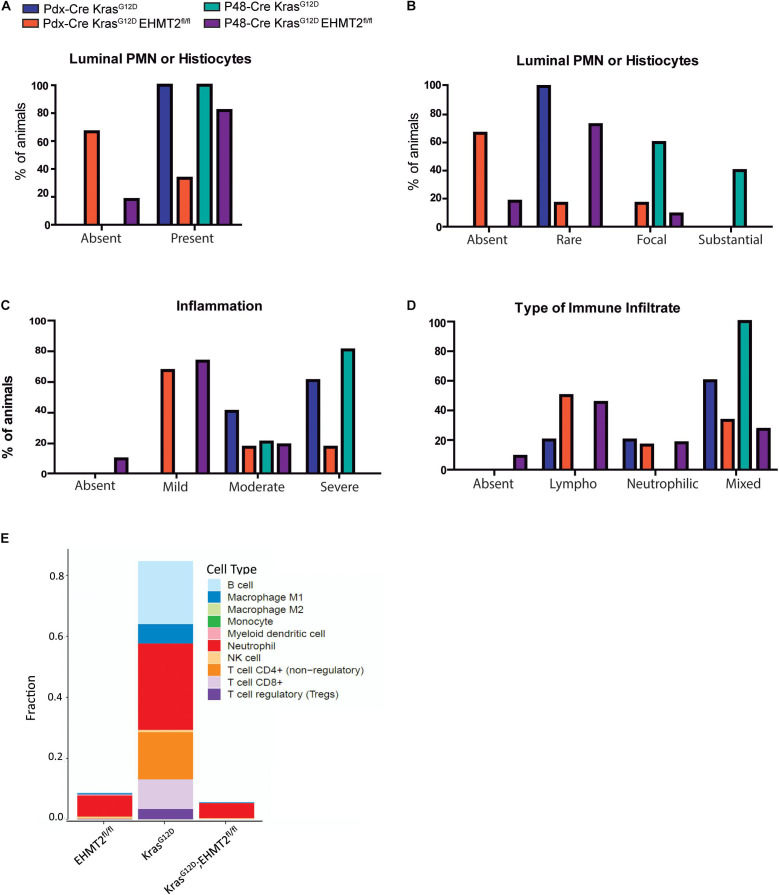
Genetic inactivation of EHMT2 in the mouse pancreas modifies the inflammatory phenotype that characterizes Kras^*G*12*D*^-mediated initiation. **(A)** Graph depicts the presence of polymorphonuclear (PMN) leukocytes or histiocytes in tubular complexes from *Kras^*G*12*D*^;EHMT2*^+/+^ and *Kras*^*G*12*D*^*;EHMT2*^*fl/fl*^ animals driven by *Pdx1-Cre* (*n* = 5 or 6, respectively) or *P48*^*Cre/+*^ (*n* = 5 or 11, respectively). **(B)** The extent of PMN or histiocyte luminal infiltration was scored as absent, rare, focal or substantial. *Kras*^*G*12*D*^ animals with *EHMT2* inactivation showed reduced infiltration of these cells for both backgrounds (*Pdx1-Cre* and *P48*^*Cre/+*^). **(C)** The degree of inflammation in tubular complexes was classified as absent, mild, moderate or severe. While *Kras*^*G*12*D*^ mice predominantly presented with moderate to severe inflammation, the majority of *Kras*^*G*12*D*^*;EHMT2*^*fl/fl*^ animals only showed mild inflammation. **(D)** The type of inflammatory cells infiltrating these precursor lesions was evaluated and classified as absent, lymphoplasmacytic (Lympho), neutrophilic or mixed. While *Kras*^*G*12*D*^ animals mostly presented with a mixed inflammatory infiltrate, *Kras*^*G*12*D*^*;EHMT2*^*fl/fl*^ mice predominantly demonstrated lymphoplasmacytic infiltrate. **(E)** Immune cell type profiling demonstrates the percentage of infiltrating immune cell types obtained by quanTIseq analysis of DEG from *Pdx1-Cre;EHMT2^*fl/fl*^*, *Pdx1-Cre;LSL-Kras^*G*12*D*^;EHMT2*^+/+^, and *Pdx1-Cre;LSL-Kras*^*G*12*D*^*;EHMT2*^*fl/fl*^ mice. Scores representing the absolute fractions of immune cells reveal that there is enrichment of T cells, neutrophils, M1-type macrophages and B cells in *Kras*^*G*12*D*^ animals, which is not present in mice with *EHMT2* inactivation.

Pancreatic Intraepithelial Neoplasia formation and progression requires infiltration of immune cells, which is known to be initiated by Kras activity (Guerra et al., 2007; di Magliano and Logsdon, 2013). In fact, an inflammatory response supports cells to overcome the oncogene-induced senescence (OIS) barrier (Guerra et al., 2011). Considering that our transcriptional signatures show an antagonistic effect of *EHMT2* inactivation on the expression of Kras-mediated inflammatory genes (Figures 5D,E), we performed histopathological examination of 8-week-old *Pdx1-Cre* pancreata to evaluate the inflammatory cell infiltration. On the *Pdx1-Cre* background, we found that 100% of *Kras*^*G*12*D*^ animals showed the presence of polymorphonuclear (PMN) leukocytes or histiocytes compared to only 33.3% of *Kras*^*G*12*D*^*;EHMT2*^*fl/fl*^ animals (Figure 7A). When evaluating *P48*^*Cre/+*^ animals, again 100% of *Kras*^*G*12*D*^ animals had infiltration of these inflammatory cells as opposed to only 81.8% of *Kras*^*G*12*D*^*;EHMT2*^*fl/fl*^ animals (Figure 7A). Subsequently, the extent of PMN or histiocyte luminal infiltration was scored as absent, rare, focal or substantial. For *Pdx1-Cre* mice, all *Kras*^*G*12*D*^ animals (100%) presented with rare infiltration, while these cells were absent in the majority of *Kras*^*G*12*D*^*;EHMT2*^*fl/fl*^ animals (66.6%) with few mice demonstrating rare (16.7%) or focal (16.7%) infiltration (Figure 7B). In a similar manner, all *P48^*Cre/+*^;Kras*^*G*12*D*^ mice showed either focal (60%) or substantial (40%) infiltration. However, in *P48*^*Cre*/+^*;Kras*^*G*12*D*^*;EHMT2*^*fl/fl*^ animals, infiltration of these cells was mostly absent (18.2%) or rare (72.7%) with a minor portion of focal (9.1%). Additionally, we examined the degree of inflammation at foci of acinar tubular complexes and atypical flat lesions, which was categorized as absent, mild, moderate or severe. All of the *Pdx1-Cre;Kras*^*G*12*D*^ animals showed moderate (40%) to severe (60%) inflammation, compared to 66.6% of the *Kras*^*G*12*D*^*;EHMT2*^*fl/fl*^ animals with only mild inflammation and few with moderate (16.7%) to severe (16.7%) inflammation (Figure 7C). Likewise, *P48^*Cre/+*^;Kras*^*G*12*D*^ animals presented with moderate (20%) to severe (80%) inflammation, whereas those carrying inactivating *EHMT2*^*fl/fl*^ alleles most commonly demonstrated mild inflammation (72.7%) with only 18.2% scored as moderate (Figure 7C). For both *Cre* backgrounds, *Kras*^*G*12*D*^ animals predominantly had a mixed cell type infiltrate (60% for *Pdx1-Cre* and 100% for *P48*^*Cre/+*^), while *Kras*^*G*12*D*^*;EHMT2*^*fl/fl*^ animals had a lymphoplasmacytic predominant cell type (50% for *Pdx1-Cre* and 45% for *P48*^*Cre/+*^), rather than neutrophilic or mixed (Figure 7D). Utilizing a deconvolution approach for bulk RNA-seq (Finotello et al., 2019), we estimated the proportions of different types of immune cell populations. This approach revealed that *Pdx1-Cre;Kras*^*G*12*D*^ animals had a markedly higher amount of DEG markers for T cells, neutrophils, B cells, and M1 macrophages (28.4, 28.3, 20.6, and 6.4%, respectively) when compared to *Pdx1-Cre;EHMT2^*fl/fl*^* (0.20, 6.98, 0.26, and 0.40%, respectively) and *Pdx1-Cre;LSL-Kras*^*G*12*D*^*;EHMT2*^*fl/fl*^ (0.06, 4.96, 0.08, and 0.27%, respectively; Figure 7E). Together, these data corroborate our gene network enrichment findings, demonstrating that *EHMT2* inactivation promotes an anti-inflammatory phenotype that antagonizes Kras^*G*12*D*^, leading to reduced inflammatory cell infiltration seen by both, histology and deconvolution of RNA-seq data.

### *EHMT2* Knockout in the Epithelium Alters the Tumor Microenvironment During Pancreatitis in *Kras* Mice

**FIGURE 8:**
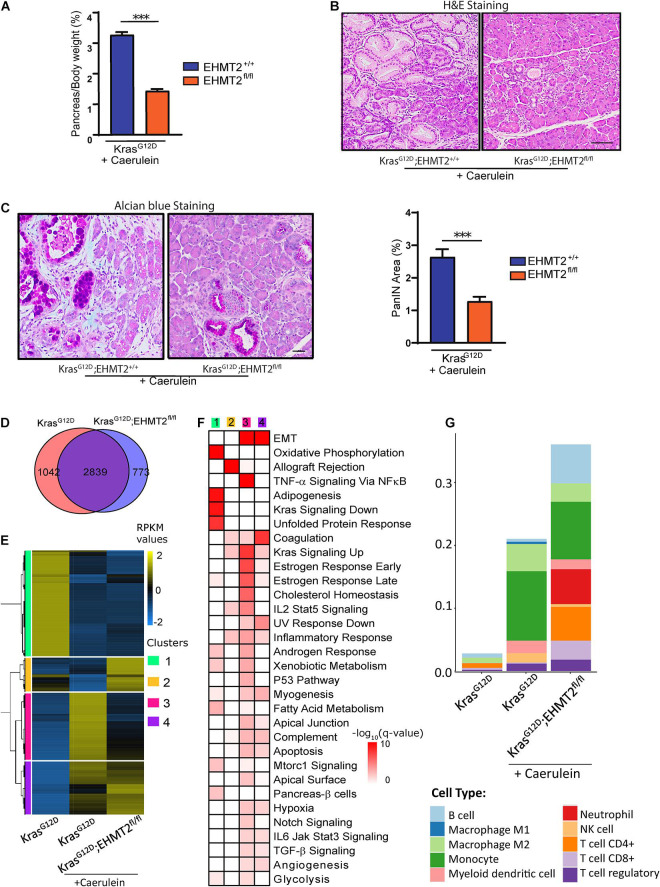
Genetic inactivation of *EHMT2* in the inflammation-accelerated Kras^*G*12*D*^ progression model diminishes effects on cell growth and gene expression. **(A)** 4-week-old *Pdx1-Cre;LSL-Kras^*G*12*D*^;EHMT2*^+/+^ (*n* = 5) and *Pdx1-Cre;LSL-Kras*^*G*12*D*^*;EHMT2*^*fl/fl*^ (*n* = 14) mice were treated with caerulein (50 μg/kg/day) for 4 weeks to induce repeated chronic pancreatitis. Graph depicts reduced pancreas-to-body weight ratios after caerulein treatment in *Pdx1-Cre;LSL-Kras*^*G*12*D*^ mice with *EHMT2* inactivation. **(B)** Representative H&E images from pancreas tissue after 4 weeks of caerulein treatment in *Pdx1-Cre;LSL-Kras^*G*12*D*^;EHMT2*^+/+^ (left) and *Pdx1-Cre;LSL-Kras*^*G*12*D*^*;EHMT2*^*fl/fl*^ (right) animals. Image on the right demonstrates significantly fewer lesions in animals carrying *EHMT2* inactivation. Scale = 50 μM. **(C)** Representative Alcian blue images from pancreas tissue after 4 weeks of caerulein treatment in *Pdx1-Cre;LSL-Kras^*G*12*D*^;EHMT2*^+/+^ and *Pdx1-Cre;LSL-Kras*^*G*12*D*^*;EHMT2*^*fl/fl*^ animals (left). Scale = 50 μM. Quantification of Alcian blue-positive PanIN lesions expressed as percentage (%) of pancreas area in *Pdx1-Cre;LSL-Kras^*G*12*D*^;EHMT2*^+/+^ (*n* = 5) and *Pdx1-Cre;LSL-Kras*^*G*12*D*^*;EHMT2*^*fl/fl*^ (*n* = 6) animals after caerulein treatment (right). **(D)** Venn diagram of DEGs in caerulein-treated *Pdx1-Cre;LSL-Kras^*G*12*D*^;EHMT2*^+/+^ and *Pdx1-Cre;LSL-Kras*^*G*12*D*^*;EHMT2*^*fl/fl*^ animals relative to age-matched, untreated *Pdx1-Cre;LSL-Kras^*G*12*D*^;EHMT2*^+/+^ control animals (*n* = 3/group). **(E)** Heatmap shows RPKM expression levels of DEGs normalized to the z-scale. Hierarchical clustering representing four major expression patterns is labeled on the left of the heatmap (green, Cluster 1; orange, Cluster 2; pink, Cluster 3; and purple, Cluster 4). Average of normalized RPKM values is represented in yellow (up), blue (down) and black (no change). **(F)** Pathway enrichment analysis of gene clusters in panel **(E)**. Color scale designates –log_10_(*q*-value) for significance of each represented pathway. Gene networks of specific pathways are illustrated in Supplementary Figure 7B. **(G)** Immune cell type profiling demonstrates the percentage of infiltrating immune cell types obtained by quanTIseq analysis of DEG from caerulein-treated *Pdx1-Cre;LSL-Kras^*G*12*D*^;EHMT2*^+/+^ and *Pdx1-Cre;LSL-Kras*^*G*12*D*^*;EHMT2*^*fl/fl*^ mice. Scores representing the absolute fractions of immune cells show that while caerulein-treated *Kras*^*G*12*D*^ animals had an enrichment in Tregs, NK cells, myeloid dendritic cells, monocytes and M2-type macrophages, caerulein-treated *Kras*^*G*12*D*^ animals with inactivation of *EHMT2* also had increased infiltration of CD8+ and non-regulatory CD4+ T cells, B cells and neutrophils. ^∗∗∗^ indicates *p*-value ≤ 0.001. All data is expressed as mean ± SEM.

Prolonged or chronic inflammatory conditions promote the initiation and development of neoplastic and fibrotic events, serving as a major risk factor leading to PDAC (Momi et al., 2012). Pancreatitis induced by caerulein, a decapeptide that stimulate Gq-coupled growth regulatory receptors (e.g., CCK), accelerates the effects of oncogenic Kras (Murtaugh and Keefe, 2015). Pancreatitis-accelerated Kras-induced neoplastic growth in mice experimentally models the inflammation-associated progression, in which the microenvironment aids the growth-promoting process (Perez-Mancera et al., 2012). We injected 4-week-old *Pdx1-Cre;LSL-Kras*^*G*12*D*^ and *Pdx1-Cre;LSL-Kras*^*G*12*D*^*;EHMT2*^*fl/fl*^ mice with caerulein for 4 weeks to induce repeated chronic pancreatitis. Macroscopic examination of the pancreas in animals sacrificed after 4 weeks of caerulein treatment demonstrated that *EHMT2* deletion antagonizes the known effect of *Kras*^*G*12*D*^ to increase pancreas-to-body weight ratios (Figure 8A; 3.27 ± 0.09% for *Pdx1-Cre;LSL-Kras*^*G*12*D*^ vs. 1.43 ± 0.06% for *Pdx1-Cre;LSL-Kras*^*G*12*D*^*;EHMT2*^*fl/fl*^; *p* < 0.001; *n* = 5 and 13, respectively). Histopathology showed extensive and dysplastic PanIN lesions with caerulein treatment in *Kras*^*G*12*D*^ animals, while pancreatic tissues from *Kras*^*G*12*D*^ mice with *EHMT2* inactivation displayed more limited dysplasia (Figure 8B). Measurements of pancreas area with mucin-rich PanIN lesions detected by Alcian Blue staining substantiated that *Kras*^*G*12*D*^*;EHMT2*^*fl/fl*^ mice had reduced area occupied by PanIN lesions, even when challenged by the inflammation-stimulating conditions (Figure 8C; 1.28 ± 0.13% *Kras*^*G*12*D*^*;EHMT2*^*fl/fl*^ + caerulein vs. 2.64 ± 0.23% *Kras*^*G*12*D*^ + caerulein; *p* < 0.001). We conclude that *EHMT2* deletion antagonizes Kras^*G*12*D*^-mediated cell growth even after enhanced stimulation by pancreatitis.

We performed RNA-seq in both caerulein-treated *Kras*^*G*12*D*^, and *Kras*^*G*12*D*^*;EHMT2*^*fl/fl*^ animals and compared them with untreated *Kras*^*G*12*D*^ mice as controls (Figure 8D; fold change ≥ |2| and FDR < 0.05). We identified 3881 caerulein-induced DEGs in *Kras*^*G*12*D*^ animals (2024 upregulated and 1857 downregulated). The upregulated subset included not only 6 distinct AP-1 transcription factors, which participate in growth, but also 19 different collagen genes and 11 chemokine ligands (Supplementary Table 5), reflecting the functional expansion of the tumor microenvironment. Among downregulated networks, we found 10 different subunits of mitochondrial respiratory chain complexes (Supplementary Table 5). Caerulein-treated *Kras*^*G*12*D*^*;EHMT2*^*fl/fl*^ mice displayed 3612 DEGs (1949 upregulated and 1663 downregulated). Of these, 2839 DEGs were shared with caerulein-treated *Kras*^*G*12*D*^ mice, but 773 DEGs were unique to *EHMT2* inactivation (Figure 8D). Thus, overall, chronic caerulein treatment of *Kras*^*G*12*D*^*;EHMT2*^*fl/fl*^ mice induces significant changes of gene expression. Consequently, we used PCA to measure genome-wide level differences in the transcriptional landscape of these animals by comparing the position of centroids in 3D (Supplementary Figure 7A). The centroid separation in 3D of the *Kras*^*G*12*D*^ and caerulein-treated *Kras*^*G*12*D*^ mice was 114, while for *Kras*^*G*12*D*^ and caerulein-treated *Kras*^*G*12*D*^*;EHMT2*^*fl/fl*^, it was 105. The distance between the two caerulein treated conditions was 60, indicative of a dominant effect of caerulein administration on gene expression. Hierarchical clustering of RPKM for these DEGs (4654 genes), depicted as a heatmap in Figure 8E, identified four main expression patterns or clusters, which were annotated for pathway enrichment analyses (Figure 8F). Cluster 1 included metabolic gene networks that were downregulated by caerulein in *Kras*^*G*12*D*^ mice regardless of *EHMT2* status (Figure 8F and Supplementary Table 6). This observation is relevant in light of the emergent relationship between metabolism and cell growth regulation by oncogenic KRAS (Kimmelman, 2015). Another important downregulated gene network, ontologically known as KRAS signaling “down,” contained genes typically downregulated by KRAS signaling (*q*-value = 6.1 × 10^–10^), which is congruent with caerulein signaling via the Gq pathway (Goldsmith and Dhanasekaran, 2007). EMT gene networks were upregulated by caerulein independent of the *EHMT2* status, forming cluster 4 (*q*-value = 1.6 × 10^–22^; Figures 7E,F and Supplementary Table 6). Cluster 2 was comprised of genes with expression upregulated by caerulein in *Kras*^*G*12*D*^*;EHMT2*^*fl/fl*^ mice in comparison with caerulein-treated *Kras*^*G*12*D*^ mice with *EHMT2* intact (Figure 8E). Networks in cluster 2 were prominently represented by immunoregulatory genes (Figure 8F and Supplementary Table 6), such as *H2-Oa* (human gene *HLA-DOA*), *H2-Ob* (human gene *HLA-DOB*), *H2-DMb2* (human gene *HLA-DMB*), *Cd2*, *Cd96*, *Cd28*, *Cd8a*, *Ccl5*, *Lck*, and *Zap70* (*q*-value = 1.5 × 10^–14^), congruent with lymphocyte infiltration of T-cell origin. Finally, cluster 3 contained genes downregulated in *Kras*^*G*12*D*^ animals carrying *EHMT2* inactivation (Figure 8E), primarily representing EMT processes (*q*-value = 7.7 × 10^–12^), TNF-α signaling via NFκB (*q*-value = 2.3 × 10^–12^), as well as genes upregulated in response to KRAS signaling (*q*-value = 1.4 × 10^–08^) among others (Figure 8F and Supplementary Table 6). Overall, these data suggest that during the process of pancreatitis-enhanced carcinogenesis by Kras^*G*12*D*^, EHMT2 influences immunomodulatory processes, while reducing EMT, TNF-α signaling *via* NFκB, and KRAS signaling, which have well-documented effects on growth promotion (Thiery et al., 2009; Taniguchi and Karin, 2018; Waters and Der, 2018). Functional inferences were further gathered by building gene expression networks represented with color-coded nodes, corresponding to fold changes in gene expression (Supplementary Figure 7B). These networks better illustrate that both, Kras and TNF-α-NFκB pathways were upregulated by pancreatitis to a significantly lesser extent in mice carrying *EHMT2* inactivation. This effect is particularly evident for a portion of the KRAS-associated gene network, composed of *Itga2*, *Etv4*, and *Wnt7a*, and the TNF-α-NFκB pathway, containing *Areg*, *Fos*, and *Cxcl5* (human gene *CXCL6*) (Supplementary Figure 7B). Notably, the growth inhibitory networks that included *Cdkn1a/p21* and *Chek2*, which were upregulated in animals with *EHMT2* inactivation under basal conditions (Figure 5F and Supplementary Figure 5), were no longer a hallmark of the caerulein-treated *Kras*^*G*12*D*^*;EHMT2*^*fl/fl*^ transcriptome. Using the same RNA-seq deconvolution approach shown in Figure 7E from pancreas tissue, we found that the treatment of *Kras*^*G*12*D*^ mice with caerulein increased regulatory T cells (Tregs), NK cells, myeloid dendritic cells, monocytes and M2 macrophages (Figure 8G). Surprisingly, caerulein-treated *Kras*^*G*12*D*^ animals with inactivation of *EHMT2* not only had the presence of the immune cell types found in the caerulein-treated *Kras*^*G*12*D*^ mice, but in addition, increased the infiltration of CD8+ and non-regulatory CD4+ T cells, B cells and neutrophils (Figure 8G). This result demonstrated that inactivation of this histone methyl transferase, in epithelial cells, leads to a change in the *Kras*^*G*12*D*^ immune landscape in response to pancreatitis by significantly enriching the infiltrate with both cytotoxic and non-regulatory T cells and B cells, which are known to work in concert to mount more efficient antitumor responses (Wörmann et al., 2014). Immunohistochemical analyses, using CD3 as a pan-T cell marker, supported the increased immune infiltration in the pancreas of caerulein-treated *Kras*^*G*12*D*^*;EHMT2*^*fl/fl*^ mice (18.76 ± 2.88% for caerulein-treated *Pdx1-Cre;LSL-Kras*^*G*12*D*^*;EHMT2*^*fl/fl*^ vs. 8.96 ± 1.66% for caerulein-treated *Pdx1-Cre;LSL-Kras*^*G*12*D*^; *p* < 0.05; *n* = 4) that was not detected in their corresponding untreated cohort (0.57 ± 0.14% for *Pdx1-Cre;LSL-Kras*^*G*12*D*^*;EHMT2*^*fl/fl*^ vs. 1.08 ± 0.28% for *Pdx1-Cre;LSL-Kras*^*G*12*D*^; *n* = 4; Supplementary Figure 7C). In summary, our data demonstrate that genetic inactivation of *EHMT2* interferes with caerulein-induced promotion of Kras^*G*12*D*^-induced effects at the gross and histopathological levels. At a molecular level, *EHMT2* inactivation affects gene expression networks involved in growth and immunoregulatory processes. This genotype-phenotype integration of transcriptomic data suggests that targeting EHMT2 for inactivation ameliorates cell growth- and inflammation-associated Kras^*G*12*D*^ functions, most likely by a combined effect not only on the targeted pancreatic epithelial cells but also in its contributions to cell populations in the microenvironment, such as those from the immune system.

## Discussion

The current study provides better insight on how the H3K9 methylation pathway, found to be altered on promoters in a specific subtype of human pancreatic cancer (Lomberk et al., 2018), influences the responses downstream of genetic alterations. This provides data to advance our understanding of the repertoire of epigenomic regulators that support the function of oncogenes (e.g., KRAS) so as to give rise to the pancreatic cancer phenotype. For PDAC, this observation extends the pathway of “initiation” from the membrane (EGFR) through the cytoplasm (KRAS) into the nucleus (EHMT2), as the functional communication between receptor and the oncogene is necessary for this process (Navas et al., 2012; Baumgart et al., 2014). Growing evidence has revealed the intricate involvement of epigenetic regulators in KRAS-mediated PDAC development, which includes those that support neoplastic progression [e.g., Bmi1 (Bednar et al., 2015) and Setdb1 (Ogawa et al., 2020)] and others serving as barriers to this process [e.g., Ezh2 (Mallen-St Clair et al., 2012), Brg1 (von Figura et al., 2014), Kdm6a (Mann et al., 2012; Andricovich et al., 2018), Arid1a (Kimura et al., 2018; Wang S. C et al., 2019; Wang W et al., 2019), Setd2 (Niu et al., 2020), and Bap1 (Perkail et al., 2020)]. Concurrent with the conclusion of our study, another report also found that *EHMT2* deficiency impairs the progression of PanIN lesions and prolongs survival of *P48*^*Cre/+*^ *Kras*^*G*12*D*^ mice (Kato et al., 2020). Our investigations corroborate and extend those findings, utilizing the *Pdx1-Cre Kras*^*G*12*D*^ mouse model in addition to the *P48*-driven Cre, to demonstrate that EHMT2 inactivation in mice with activated Kras^*G*12*D*^ inhibits ADM and PanIN formation. By inactivating EHMT2 in the pancreas with either *Pdx1-Cre* or *P48*^*Cre/+*^, we provide robust evidence that this pathway is not required for pancreas exocrine development and is tolerated in this organ under basal contexts. While EHMT2 is critical to support mouse embryonic life at the level of the whole organism (Tachibana et al., 2002), we add the pancreas to the list of cell lineages, such as skeletal muscle (Zhang et al., 2016), that do not require this epigenetic regulator for proper development. Setdb1, a methyltransferase for the H3K9me3 mark, was also recently shown to also be dispensable for proper pancreas development in mice (Ogawa et al., 2020). Thus, H3K9 methylation pathways, at least after embryonic day 8.5 or 9.5, do not appear to be necessary during pancreatic development, although it remains unknown whether confounding effects would occur with more than one H3K9 methyltransferase inactivated simultaneously. Genetic inactivation of *EHMT2* with the same two Cre-drivers crossed to the *LSL-Kras*^*G*12*D*^ model and the tamoxifen-inducible *CAGGCre-ER*^TM^ demonstrated how loss of this epigenetic complex antagonizes EGF-KRAS-mediated PDAC initiation via ADM, *in vitro* and *in vivo*, as well as PanIN formation *in vivo*. Furthermore, we show that levels of the EHMT2-EHMT1-WIZ complex in exocrine pancreatic cells are normally low but are induced by the KRAS growth regulatory pathway. This increase in formation of the enzymatic EHMT2-EHMT1-WIZ complex in response to KRAS leads to enhanced deposition of its product, the H3K9me2 mark, supporting a novel role of this epigenetic regulator downstream of this mitogenic signaling pathway.

To examine molecular mechanisms that may account for how EHMT2 inactivation antagonizes the functions of oncogenic Kras^*G*12*D*^, we considered the properties of this epigenomic protein on transcriptional regulation. Indeed, RNA-seq and targeted pathway-specific RT-qPCR analyses indicated that mice carrying conditional *EHMT2* inactivation in their pancreas had transcriptional profiles that were dominant over the Kras^*G*12*D*^-regulated gene expression program, highlighting the contribution that this histone methyltransferase has in promoting the Kras^*G*12*D*^-regulated gene expression program. *EHMT2* inactivation resulted in the upregulation of key cell cycle inhibitory checkpoints, including *Chk2* and *Cdkn1a/p21*, which function in cell cycle arrest in a manner that if persistent can induce senescence (Aliouat-Denis et al., 2005; d’Adda di Fagagna, 2008). Congruently, investigations of cellular mechanisms that could be responsible for the antagonism of Kras^*G*12*D*^-mediated growth determined that *EHMT2* deletion in the *Kras*^*G*12*D*^-expressing exocrine pancreas leads to senescence. Senescence is mechanistically important since it occurs in the context of Kras^*G*12*D*^ oncogene-induced stress, a phenomenon that is operational in pancreatic cells and drives the accelerated firing of replication forks with uninterrupted cycles of cell proliferation caused by the oncogenic stimulus (Kotsantis et al., 2018). Under normal conditions, this stress is compensated so that this oncogene can proceed with accelerated growth unless cell cycle regulators and checkpoint proteins become activated leading to OIS (Liu et al., 2018). OIS is often a dominant mechanism that antagonizes the transformation process (Lowe et al., 2004). However, studies have shown that physiological levels of oncogenic Kras have the capacity to suppress premature senescence of pancreatic ductal epithelium *in vivo*, bypassing this process (Lee and Bar-Sagi, 2010). In this regard, our data is, at least in large part, consistent with the escape from senescence being operational under *Kras*^*G*12*D*^ activation *in vivo*, which is thwarted when combined with *EHMT2* deletion. Indeed, combined data from histology, IHC, β-galactosidase staining, RT-qPCR and RNA-seq identifies the previously unknown cooperation between EHMT2 and Kras^*G*12*D*^, which must be actively maintained to eventually bypass OIS and support PDAC initiation. Inactivation of EHMT2 in other organs can trigger different mechanisms, depending upon the cell type and the physiological or pathological context (Shankar et al., 2013; Casciello et al., 2015; Kramer, 2015). In these studies, EHMT2 has been found to participate in a large number of phenomena, and in some cells, loss of this epigenetic regulator induces senescence even in the absence of oncogenic stimulation (Takahashi et al., 2012; Wang et al., 2013). Interestingly, ectopic oncogenic Ras-induced senescence in human diploid fibroblasts results in proteosomal degradation of the EHMT2 complex by APC/C(cdh1) (Takahashi et al., 2012). In addition, pharmacological inhibitors of EHMT2, which have similar pleomorphic effects in distinct cells and tissues (Casciello et al., 2015; Charles et al., 2019; Griñán-Ferré et al., 2019; Kim et al., 2019; Rybak et al., 2019), in certain cases, also induce senescence (Yuan et al., 2012). Altogether, our results support the conclusion that enhanced senescence is one important cellular mechanism by which EHMT2 inactivation appears to antagonize Kras^*G*12*D*^ in exocrine pancreatic cells *in vivo*. Notably, the effects of EHMT2 inactivation on Kras^*G*12*D*^-induced growth remained under pancreatitis-stimulated conditions, providing further evidence that targeting this epigenomic regulator exerts a dominant effect over the functions of this oncogene. However, we also found that *EHMT2* inactivation in caerulein-treated Kras^*G*12*D*^ mice had additional impact on expression of immunoregulatory gene networks, which were not activated by the genetic manipulation of either *EHMT2* or *Kras*^*G*12*D*^ alone, and altered the composition of immune cell infiltration, enriching the proportion of cytotoxic and non-regulatory T cells and B cells, which is suggestive of an antitumor response (Wörmann et al., 2014). Thus, EHMT2 inactivation appears play a role in regulating pancreatitis-enhanced Kras^*G*12*D*^ effects, not only through cell growth regulatory pathways, but also in part *via* immunomodulatory effects, thereby affecting the tumor microenvironment.

In summary, this work identifies EHMT2 as a KRAS-inducible epigenetic regulator which enables this oncogene to exert its effects on growth and inflammation, even when challenged in the pancreatitis-associated promotion model. Indeed, at the molecular level Kras^*G*12*D*^ induces the levels of EHMT2, its heterotrimeric complex with EHMT1 and WIZ, as well as its enzymatic product, H3K9me2. Consequently, EHMT2 inactivation significantly reduces the levels of H3K9me2. The role of EHMT2 as a regulator of gene expression is clearly demonstrated by the fact that its inactivation changes the transcriptional profile of Kras^*G*12*D*^ in a dominant manner. Noteworthy, while we manipulated the KRAS-EHMT2 pathway in epithelial cells, the results extend beyond this compartment to affect the tissue microenvironment, namely immune cell populations.

Besides the mechanistic importance of these results, this new information reinforces the role of EHMT2 as a potential therapeutic or chemopreventive target for pancreatic cancer and highlights the possibilities of this therapeutic strategy in combination with current inhibitors of the EGFR-KRAS pathway, which are widely available for clinical trials. In addition, the discovery that inhibition of EHMT2 in epithelial cells leads to reorganization of the immune landscape in the tumor microenvironment serves as the foundation for future studies focused on designing combinations with immunomodulatory agents. Lastly, the existence of this novel KRAS-EHMT2 pathway that is critical for mediating the growth-promoting and immunoregulatory effects of this oncogene *in vivo* predicts that these therapies will likely impact both the tumor-initiating epithelial cells and the tumor microenvironment.

## Data Availability Statement

The datasets presented in this study can be found in online repositories. The names of the repository/repositories and accession number(s) can be found below: GEO with dataset number GSE169525.

## Ethics Statement

Animal care and all protocols were reviewed and approved by the Institutional Animal Care and Use Committees of Mayo Clinic Rochester (IACUC protocols A00002240-16 and A24815) and the Medical College of Wisconsin (AUA00005963).

## Author Contributions

RU and GL conceived and designed the study. GU, TA, AM, and AS conducted the experiments, acquired and analyzed the data. GU, RK, AZ, TS, VA, and BP participated in formal analysis. TA assembled figures. GU, TA, AM, MBD, MZ, JI, RU, and GL contributed to data interpretation. TA, JI, RU, and GL wrote the original draft. GL supervised the study. All authors provided valuable intellectual input on experiments, as well as read, offered feedback and approved the manuscript.

## Conflict of Interest

MBD is a co-founder and has ownership interests in Protein Foundry, LLC and Xlock Biosciences, LLC. The remaining authors declare that the research was conducted in the absence of any commercial or financial relationships that could be construed as a potential conflict of interest.
