# Supplementary material for: Inactivation of the Euchromatic Histone-Lysine N-Methyltransferase 2 Pathway in Pancreatic Epithelial Cells Antagonizes Cancer Initiation and Pancreatitis-Associated Promotion by Altering Growth and Immune Gene Expression Networks
Source: Front Cell Dev Biol. 2021 Jun 23;9:681153. doi: 10.3389/fcell.2021.681153 (PMC8261250; doi:10.3389/fcell.2021.681153)
Supplement: Supplementary file 1 [file Data_Sheet_1.pdf]

Supplementary Material

for

Inactivation of the Euchromatic Histone-lysine *N*-methyltransferase 2 Pathway in Pancreatic Epithelial Cells Antagonizes Cancer Initiation and Pancreatitis-Associated Promotion by Altering Growth and Immune Gene Expression Networks

Guillermo Urrutia[1†], Thiago Milech de Assuncao[1,2,†], Angela J. Mathison[1,2], Ann Salmonson[1], Romica Kerketta[1,2], Atefeh Zeighami[1,2], Timothy J. Stodola[1,2], Volkan Adsay[3], Burcin Pehlivanoglu[4], Michael B. Dwinell[1,5,6,7], Michael T. Zimmermann[2,8,9], Juan L. Iovanna[10], Raul Urrutia[1,2,7,8,11,*], Gwen Lomberk[1,2,7,12,*]

Supplementary Figure 1

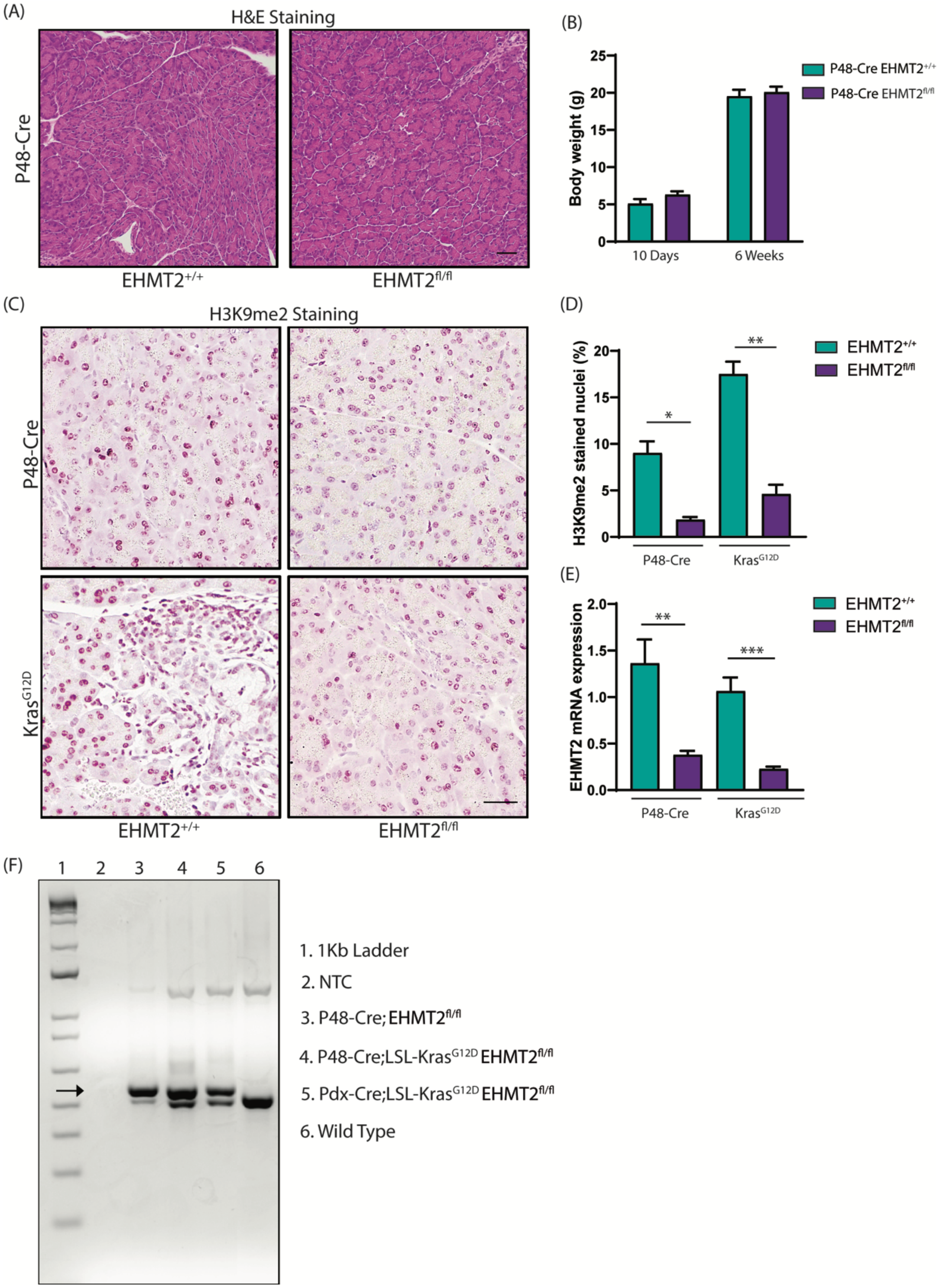

Supplementary Figure 1. *Conditional knockout of EHMT2 driven by P48$^{Cre/+}$ does not alter normal mouse pancreas development or architecture.*

(A) Representative H&E images of pancreas tissue from *P48$^{Cre/+}$;EHMT2$^{+/+}$* and *P48$^{Cre/+}$;EHMT2$^{fl/fl}$* mice. No significant histological or architectural changes were observed. Scale= 50µM. **(B)** Graph

illustrates body weight of animals at 10 days (*n*= 5 *P48*$^{Cre/+}$;*EHMT2*$^{+/+}$ and *n*= 3 *P48*$^{Cre/+}$;*EHMT2*$^{fl/fl}$) and 6 weeks (*n*= 6 *P48*$^{Cre/+}$;*EHMT2*$^{+/+}$ and *n*= 14 *P48*$^{Cre/+}$;*EHMT2*$^{fl/fl}$) of age. **(C)** Representative images from H3K9me2 IHC on pancreas tissue from *P48*$^{Cre/+}$;*EHMT2*$^{+/+}$ and *P48*$^{Cre/+}$;*EHMT2*$^{fl/fl}$ mice, as well as *P48*$^{Cre/+}$*;LSL-Kras*^{G12D}*;EHMT2*$^{+/+}$ and *P48*$^{Cre/+}$*;LSL-Kras*^{G12D}*;EHMT2*$^{fl/fl}$ animals. Scale= 50μM. **(D)** Percentage of nuclei positive for H3K9me2 was quantified from a minimum of 5 random fields at 10X magnification (*n*= 3/group). **(E)** Levels of *EHMT2* transcript were evaluated by RT-qPCR on RNA of whole pancreas isolated from *P48*$^{Cre/+}$;*EHMT2*$^{+/+}$ and *P48*$^{Cre/+}$;*EHMT2*$^{fl/fl}$ mice, as well as *P48*$^{Cre/+}$*;LSL-Kras*^{G12D}*;EHMT2*$^{+/+}$ and *P48*$^{Cre/+}$*;LSL-Kras*^{G12D}*;EHMT2*$^{fl/fl}$ animals (*n*= 8/group). **(F)** PCR for *EHMT2* exon excision was performed on non-template control (NTC) or whole pancreas DNA from *P48*$^{+/-Cre}$;*EHMT2*$^{fl/fl}$, *P48*$^{Cre/+}$*;LSL-Kras*^{G12D}*;EHMT2*$^{fl/fl}$ and *Pdx1-Cre;LSL-Kras*^{G12D}*;EHMT2*$^{fl/fl}$ mice. A 500bp band represents *EHMT2* exon excision (arrow) and a 450bp band represents the wild-type allele. * indicates p-value ≤ 0.05, ** indicates p-value ≤ 0.01, and *** indicates p-value ≤ 0.001. All graphed data is expressed as mean ± SEM.

Supplementary Figure 2

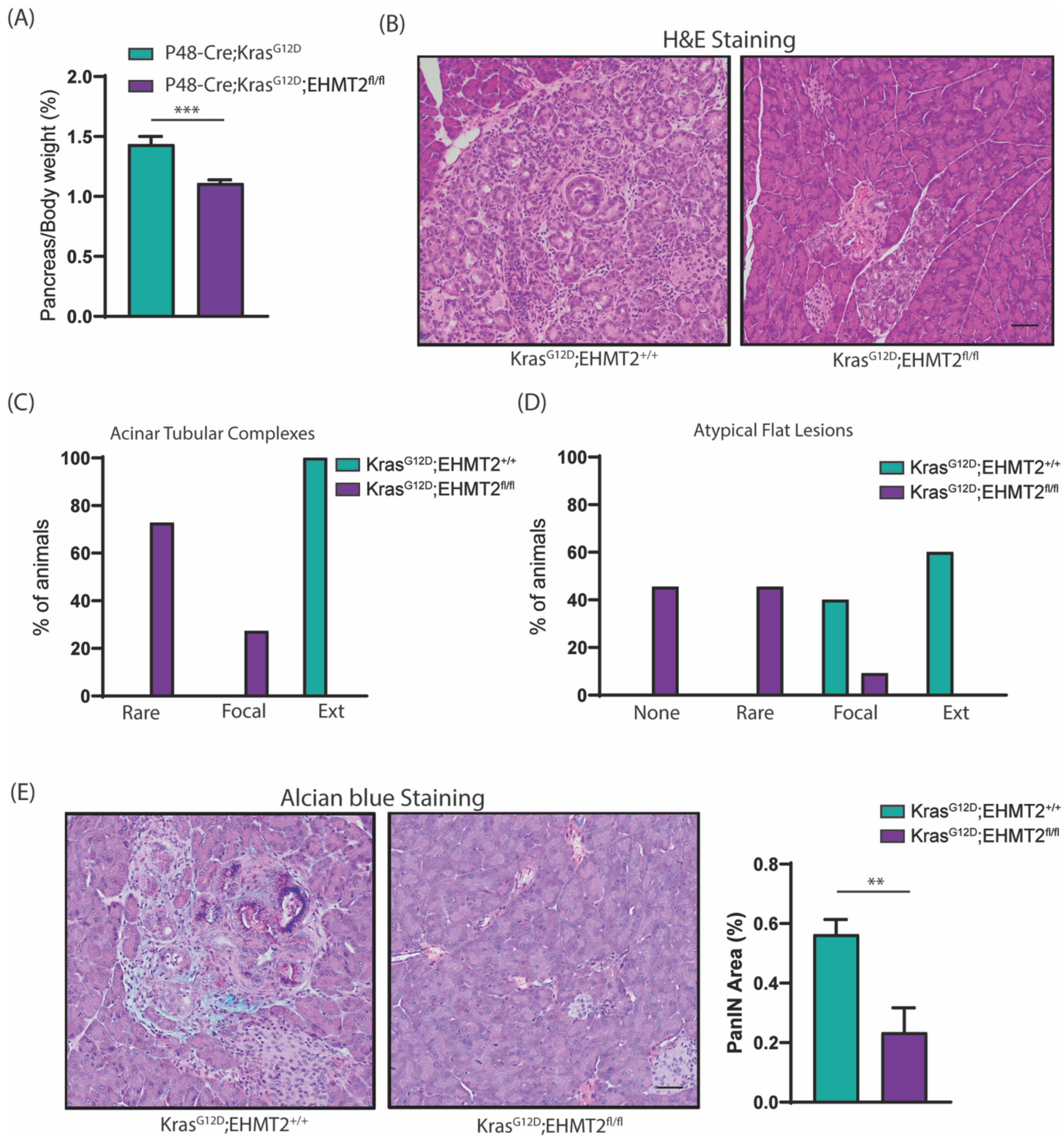

Supplementary Figure 2. *Genetic inactivation of EHMT2 also antagonizes the increased pancreas-to-body weight ratios and PanIN development induced by the P48$^{Cre/+}$-driven Kras^{G12D} mouse model.*

(A) Graph demonstrates reduced pancreas-to-body weight ratios after *EHMT2* inactivation in *P48$^{Cre/+}$;LSL-Kras^{G12D}* mice (*n*= 13) compared to *P48$^{Cre/+}$;LSL-Kras^{G12D};EHMT2$^{+/+}$* animals (*n*= 8). **(B)** Representative images of H&E stained pancreas tissue from *P48$^{Cre/+}$;LSL-Kras^{G12D};EHMT2$^{+/+}$* (left) and *P48$^{Cre/+}$;LSL-Kras^{G12D};EHMT2$^{fl/fl}$* (right) animals. Image on the right shows fewer PanIN lesions after *EHMT2* inactivation. Scale= 50μM. Graph depicts scoring from histological assessment of precursor acinar tubular complexes **(C)** and atypical flat lesions **(D)** in pancreatic tissue from

$P48^{Cre/+}$;LSL-$Kras^{G12D}$;$EHMT2^{+/+}$ (*n*= 5) and $P48^{Cre/+}$;LSL-$Kras^{G12D}$;$EHMT2^{fl/fl}$ (*n*= 11) mice. Lesions were classified as none (for atypical flat lesions only), rare, focal or extensive (Ext). **(E)** Alcian blue staining was used to quantify the burden of mucin rich PanIN lesions. Left: Representative images from Alcian blue staining on $P48^{Cre/+}$;LSL-$Kras^{G12D}$;$EHMT2^{+/+}$ and $P48^{Cre/+}$;LSL-$Kras^{G12D}$;$EHMT2^{fl/fl}$ pancreas tissue. Scale= 50μM. Left: quantification of Alcian blue-positive PanIN lesions expressed as percentage (%) of pancreas area from $P48^{Cre/+}$;LSL-$Kras^{G12D}$;$EHMT2^{+/+}$ (left, *n*= 7) and $P48^{Cre/+}$;LSL-$Kras^{G12D}$;$EHMT2^{fl/fl}$ (right, *n*= 6) animals. * indicates p-value ≤ 0.05, and *** indicates p-value ≤ 0.001. All graphed data is expressed as mean ± SEM.

Supplementary Figure 3

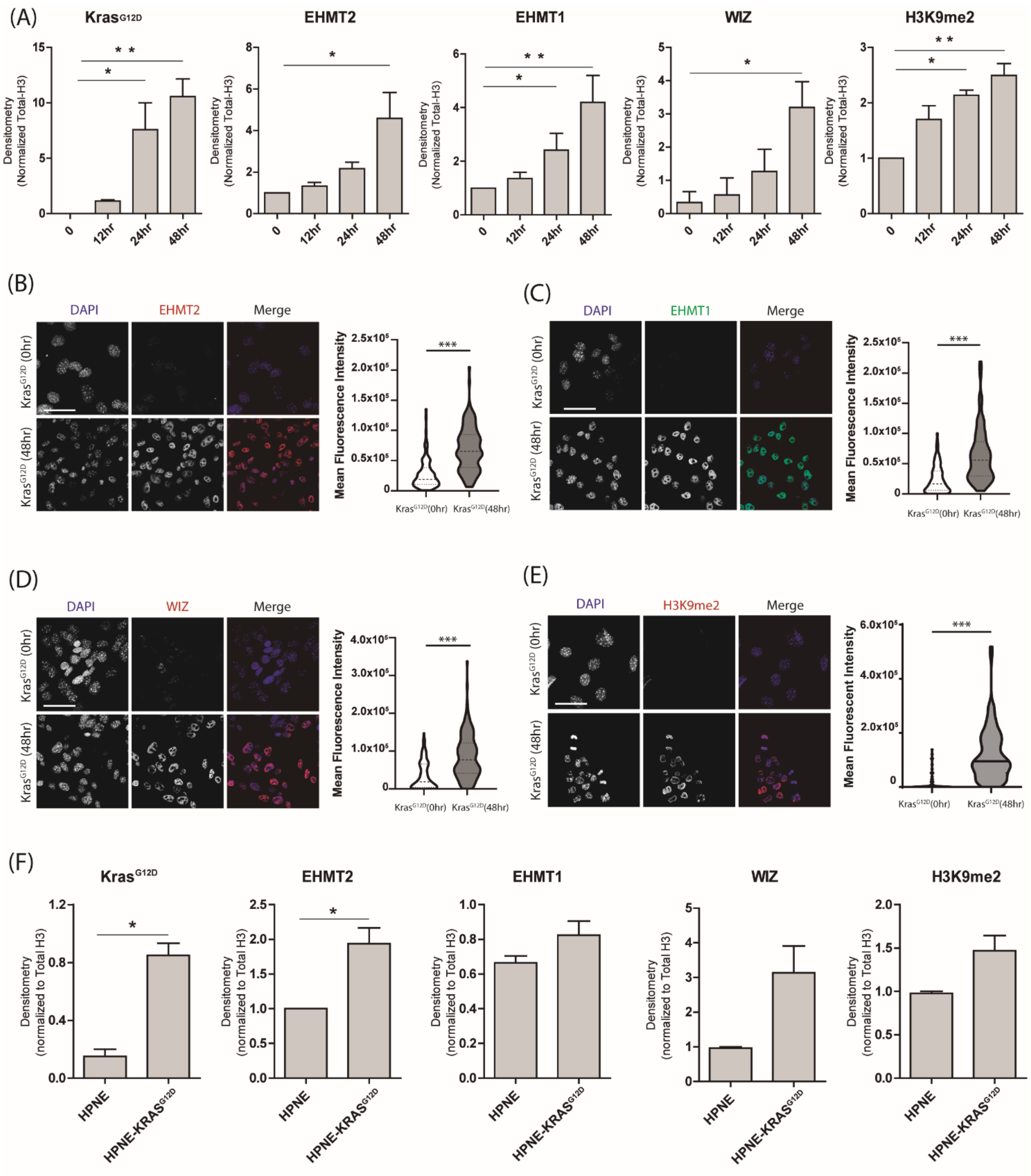

(G)

Protein	Control peptide count		Target peptide count		Score
	IgG 0hr	IgG 24hr	EHMT2 0hr	EHMT2 24hr	
EHMT1	0	0	0	6.3053	100%
EHMT2	0	0	0.8334	16.394	90%
WIZ	0	0	0.8334	6.3053	77%

Supplementary Figure 3. *$Kras^{G12D}$ expression increases levels of the EHMT2-EHMT1-WIZ complex and H3K9me2 deposition.*

(A) Densitometric quantification of Western blot assays performed from iKras 4292 cell lysates in Figure 4A. Graphs depict time-dependent increase of target proteins after $Kras^{G12D}$ expression (experiment performed in triplicate). Cells were stimulated with doxycycline 12, 24 and 48 hr to induce $Kras^{G12D}$, then expression of EHMT2, EHMT1, WIZ, $Kras^{G12D}$ and H3K9me2 was evaluated using Total H3 as loading control. **(B)** Immunofluorescence-based microscopy of EHMT2 on iKras 4292 cells after 0 and 48 hr of $Kras^{G12D}$ expression (left). Violin plot representing Mean Fluorescence Intensity (MFI) of EHMT2 signal (right, experiment performed in triplicate). **(C)** Immunofluorescence-based microscopy of EHMT1 on iKras 4292 cells after 0 and 48 hr of $Kras^{G12D}$ expression (left). Violin plot representing MFI of EHMT1 signal (right, experiment performed in triplicate). **(D)** Immunofluorescence-based confocal microscopy of WIZ on iKras 4292 cells after 0 and 48 hr of $Kras^{G12D}$ expression (left). Violin plot representing MFI of WIZ signal (right, experiment performed in triplicate). **(E)** Immunofluorescent staining of H3K9me2 on iKras 4292 cells after 0 and 48 hr of $Kras^{G12D}$ expression (left). Violin plot representing MFI of H3K9me2 signal (right). Scale= 50µM. **(F)** Densitometric quantification of Western blot assays performed from HPNE and HPNE-$KRAS^{G12D}$ cell lysate in Figure 4B. Graphs show higher expression of target proteins when HPNE cells are compared to HPNE-$KRAS^{G12D}$ (experiment performed in triplicate). Expression of $KRAS^{G12D}$, EHMT2, EHMT1, WIZ and H3K9me2 was evaluated using Total H3 as loading control. **(G)** Table of results from immunoprecipitation of EHMT2 from iKras 4292 cells followed by mass spectrometry analysis. Normalized total spectral counts are shown for immunoprecipitations with IgG control and EHMT2 antibodies at both, 0hr and 24hr post $Kras^{G12D}$ expression. Enrichment score is shown for EHMT2 complexes at 24hr of $Kras^{G12D}$ expression, using the formula: *(total of peptides 24h – total peptides 0h) / (total of peptides 24h + total peptides 0h)*. * indicates p-value ≤ 0.05 and *** indicates p-value ≤ 0.001; Student´s T-test. All graphed data is expressed as mean ± SEM.

Supplementary Figure 4

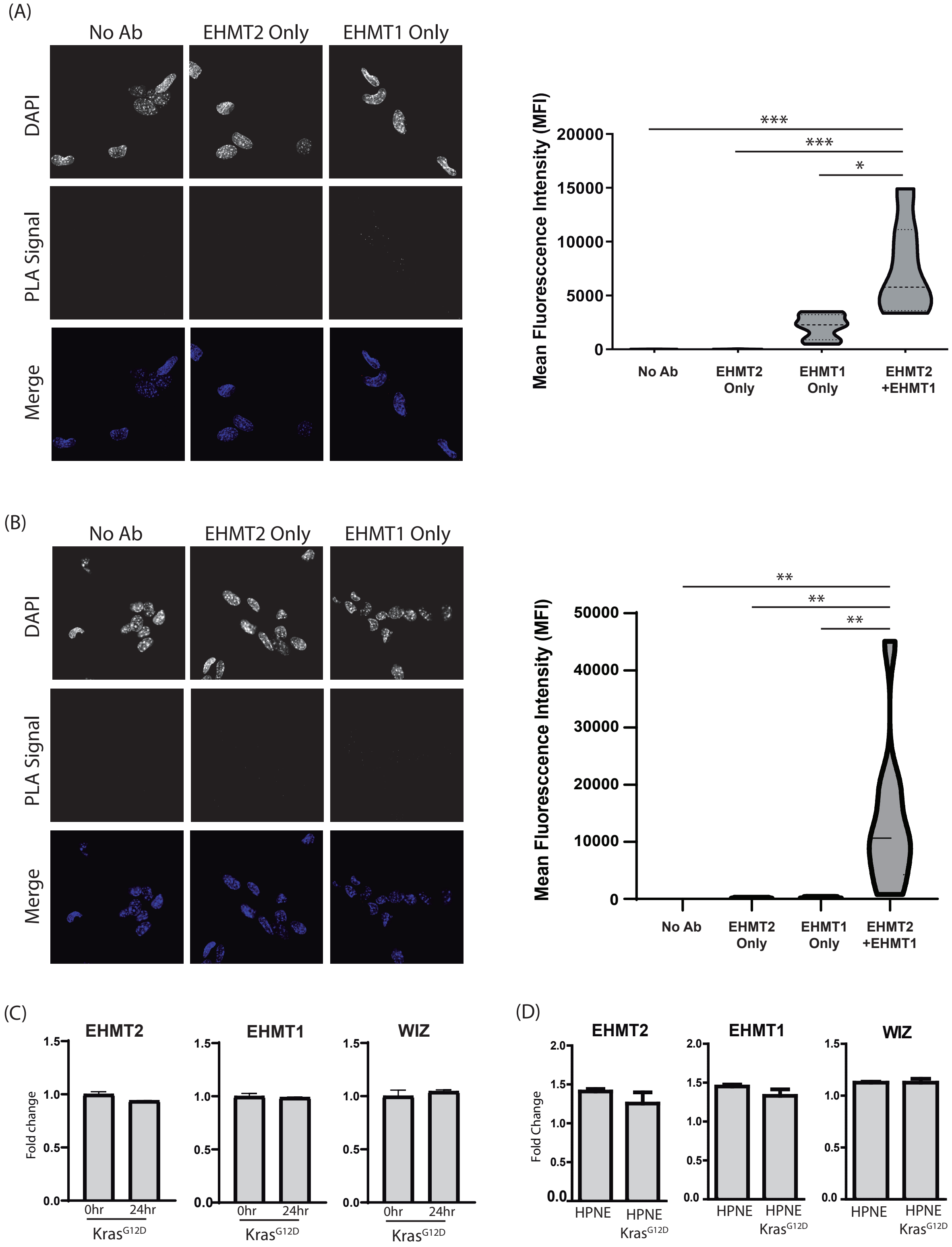

Supplementary Figure 4. *$Kras^{G12D}$ expression increases members of the EHMT2 complex at the protein level and their interaction without altering transcript levels.*

(A) EHMT2-EHMT1 interaction by PLA was evaluated with individual controls. Left: Representative images of iKras 4292 cells at 24 hr post $Kras^{G12D}$ activation using no antibody or individual EHMT2 or EHMT1 antibodies for PLA reaction to control specificity shown in Figure 1c. Scale= 50μM. Right: Violin plot representing Mean Fluorescence Intensity (MFI) of No antibody, EHMT2 alone, and EHMT1 alone compared to positive EHMT2+EHMT1 PLA signal (experiment performed in triplicate). **(B)** EHMT2-WIZ interaction by PLA was evaluated with individual controls. Left: Representative images of iKras 4292 cells at 24 hr post $Kras^{G12D}$ activation using no antibody or individual EHMT2 or WIZ antibodies for PLA reaction to control specificity shown in Figure 4D. Scale= 50μM. Right: Violin plot representing Mean Fluorescence Intensity (MFI) of No antibody, EHMT2 alone, and WIZ alone compared to positive EHMT2+WIZ PLA signal (experiment performed in triplicate). **(C)** Fold change from RPKM counts of *EHMT2*, *EHMT1* and *WIZ* are shown from iKras 4292 cells after 0 and 48 hr of $Kras^{G12D}$ expression by RNA-seq (n= 3). **(D)** Fold change from mRNA levels of *EHMT2*, *EHMT1* and *WIZ* were evaluated on HPNE and HPNE-$KRAS^{G12D}$ by RT-qPCR (n= 3). * indicates p-value ≤ 0.05, ** indicates p-value ≤ 0.01, and *** indicates p-value ≤ 0.001; Student's T-test. All data is expressed as mean ± SEM.

Supplementary Figure 5

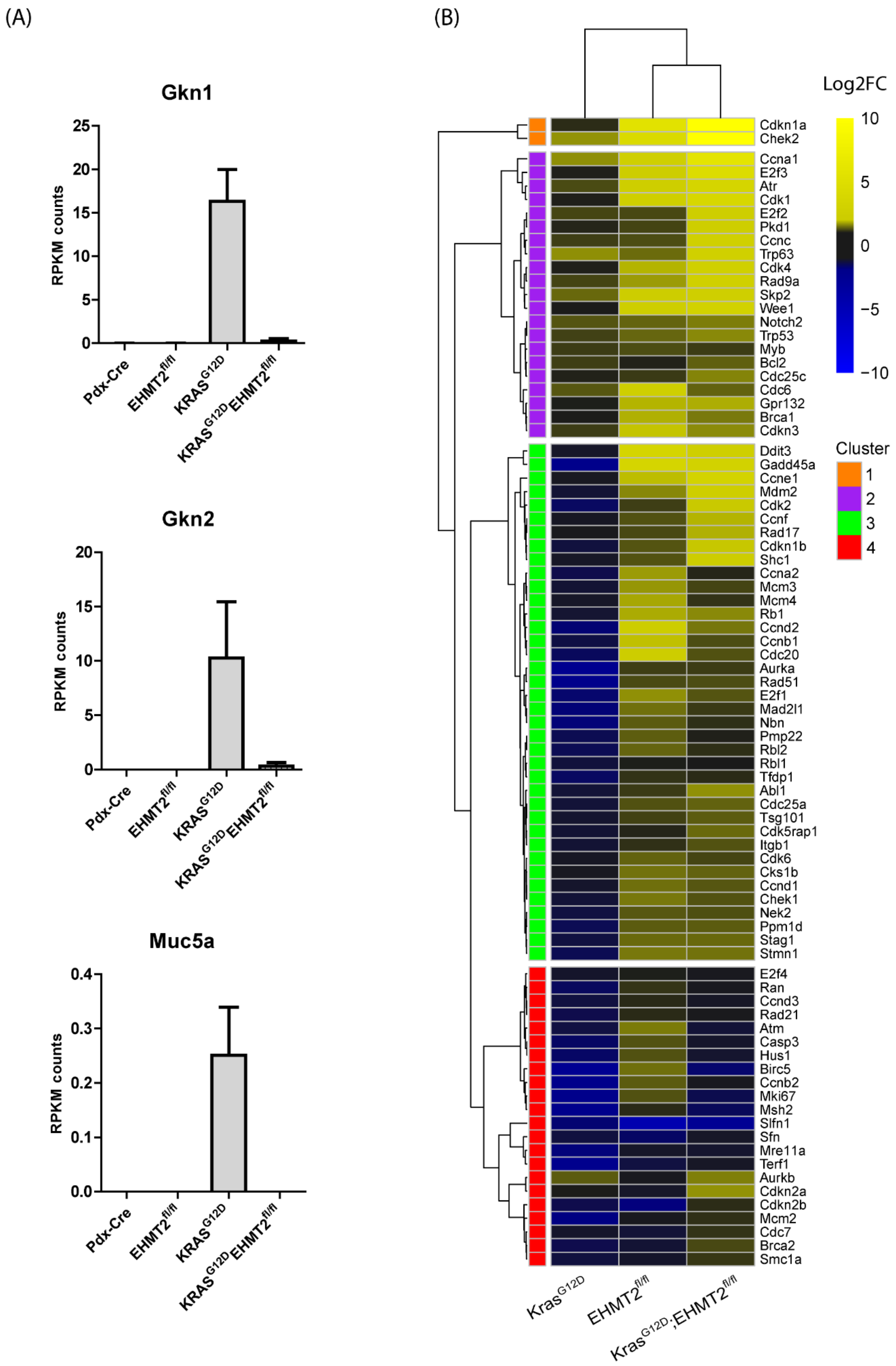

Supplementary Figure 5. *EHMT2 inactivation in the pancreas downregulates PanIN markers and upregulates genes involved in cell cycle arrest after DNA damage, which contribute to a transcriptional response that antagonizes KRAS-mediated proliferative stress*.

(A) Total RPKM counts of *Gkn1*, *Gkn2* and *Muc5a* are shown from *Pdx1-Cre, Pdx1-Cre;EHMT2*$^{fl/fl}$, *Pdx1-Cre;LSL-Kras*^{G12D}*;EHMT2*$^{+/+}$ and *Pdx1-Cre;LSL-Kras*^{G12D}*;EHMT2*$^{fl/fl}$ expression by RNA-seq **(B)** Heatmap shows fold change levels for 83 cell cycle-related genes in *Pdx1-Cre;EHMT2*$^{fl/fl}$, *Pdx1-Cre;LSL-Kras*^{G12D}*;EHMT2*$^{+/+}$ and *Pdx1-Cre;LSL-Kras*^{G12D}*;EHMT2*$^{fl/fl}$ mice relative to *Pdx1-Cre* control animals, as determined by RT-qPCR (*n*= 3/group). Log2FC relative to *Pdx1-Cre* are represented in yellow (up), blue (down) and black (no change). Hierarchical clustering revealed four main expression patterns, as labeled on the left of the heatmap (orange, Cluster 1; purple, Cluster 2; green, Cluster 3; and red, Cluster 4). Cluster 1 harbored genes with the largest fold change as a result of EHMT2 inactivation, namely *Cdkn1a/p21* and *Chek2*.

Supplementary Figure 6

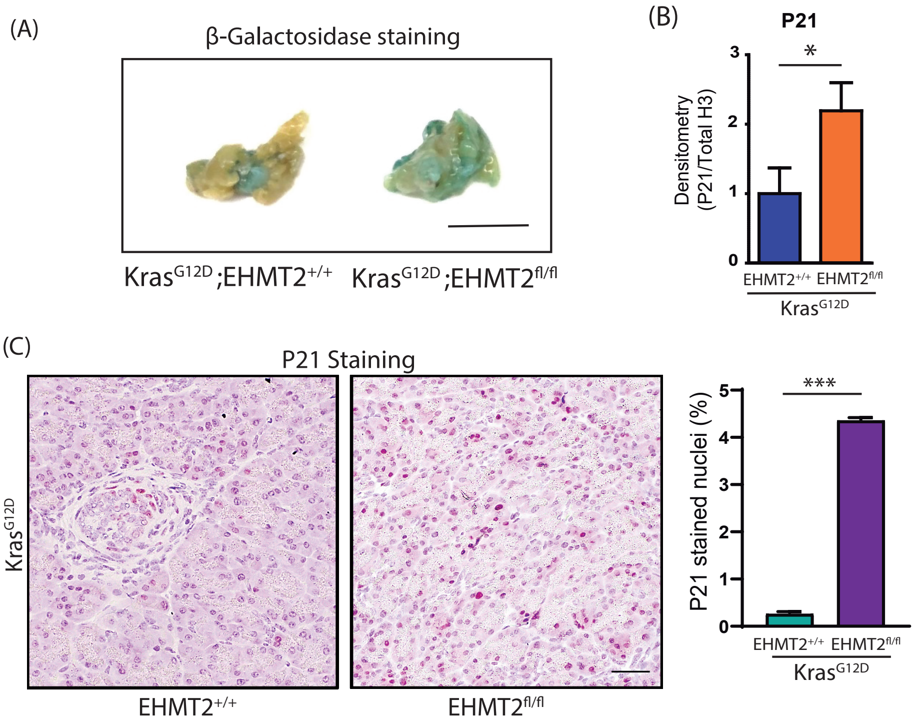

Supplementary Figure 6. *EHMT2 inactivation also induces cell senescence in the P48$^{Cre/+}$-driven Kras^{G12D}-expressing mouse pancreas coincident with increased P21.*

(A) Senescence-associated β-galactosidase staining on fresh pancreas tissue extracted from *P48$^{Cre/+}$;LSL-Kras^{G12D};EHMT2$^{+/+}$* and *P48$^{Cre/+}$;LSL-Kras^{G12D};EHMT2$^{fl/fl}$* animals. The presence of blue staining indicates senescence. Scale= 1mm. **(B)** Graph shows quantification of densitometry from Western blot evaluation of P21 expression shown in Figure 6B, demonstrating an increase in P21 protein levels in *Pdx1-Cre;LSL-Kras^{G12D};*EHMT2$^{fl/fl}$ animals. Total-H3 was used as loading control to normalize the samples. **(C)** Left: Representative images from IHC staining for P21 on pancreas tissues from *P48$^{Cre/+}$;LSL-Kras^{G12D};EHMT2$^{+/+}$* and *P48$^{Cre/+}$;LSL-Kras^{G12D};EHMT2$^{fl/fl}$* animals. Scale= 50μM. Right: Graph represents percentage of nuclei positive for P21 as quantified from a minimum of 5 random fields at 10X magnification, (*n*= 3/group). *** indicates p-value $\leq$ 0.001; Student´s T-test with Welch's correction. All graphed data is expressed as mean ± SEM.

Supplementary Figure 7

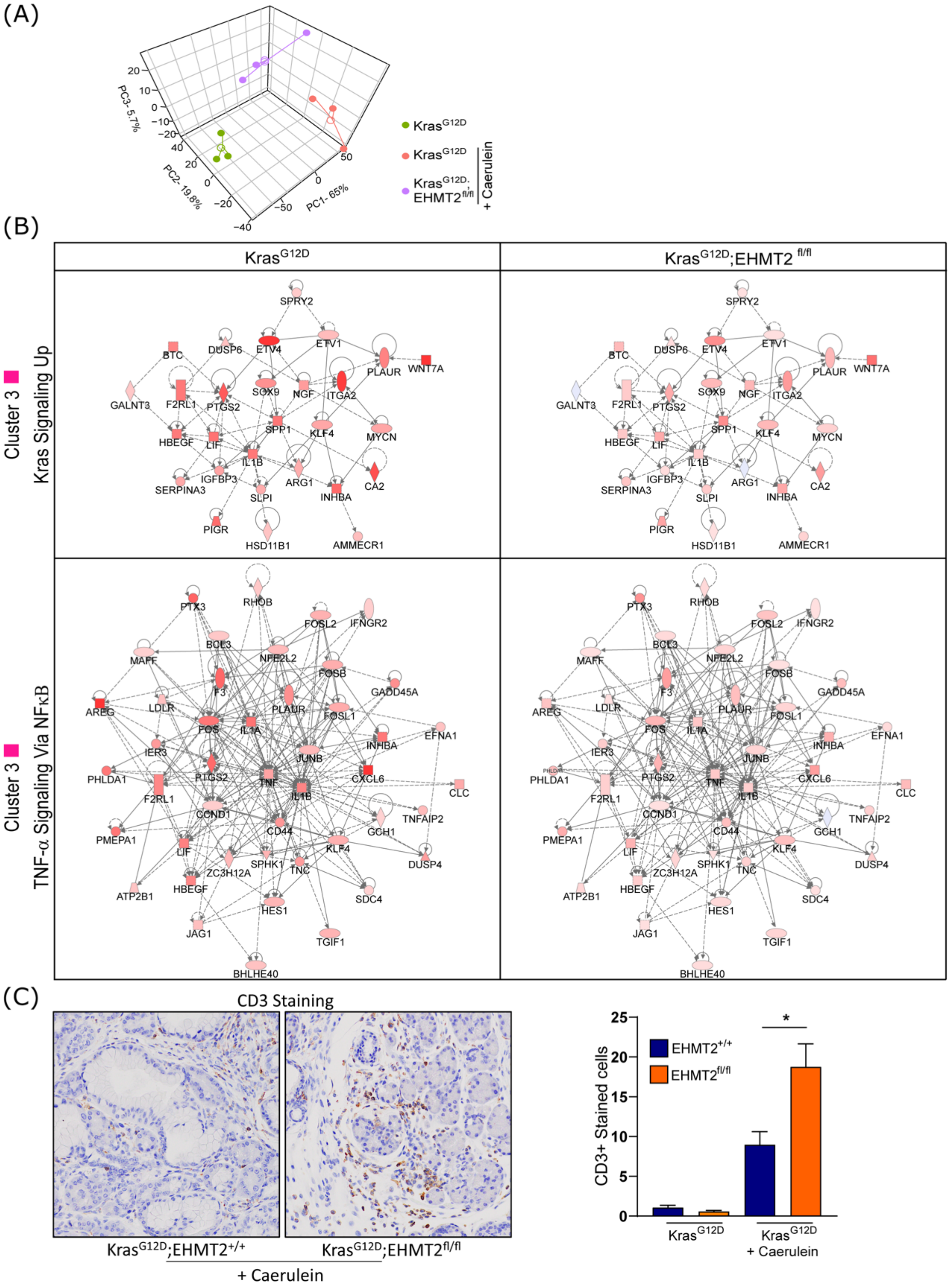

Supplementary Figure 7. *PCA plot and network analysis of pathways affected by EHMT2 inactivation in the caerulein-stimulated Kras^{G12D} acceleration model.*

(A) PCA plot comparing different groups with centroids (open circles) and individual animals in each group (filled circles) using RPKM expression values of DEGs. **(B)** Gene networks of specific pathways enriched in Cluster 3 of heatmap in Figure 8E, which contained genes upregulated in caerulein-treated *Kras^{G12D}* animals and relatively less expressed in caerulein-treated *Kras^{G12D};EHMT2$^{fl/fl}$* animals. Gene fold changes with respect to untreated control Kras^{G12D} animals are represented with red (up) and blue (down) nodes. **(C)** Left: Representative IHC images of CD3+ cells on pancreatic tissue from *Pdx1-Cre;LSL-Kras^{G12D};EHMT2$^{+/+}$* and *Pdx1-Cre;LSL-Kras^{G12D};EHMT2$^{fl/fl}$* animals treated with caerulein. Right: Graph represents percentage of CD3 positive cells as quantified from a minimum of 10 random fields at 10X magnification, (*n*= 4/group). * indicates p-value ≤ 0.05; Student´s T-test. All graphed data is expressed as mean ± SEM.
